# The zebrafish progranulin gene family and antisense transcripts

**DOI:** 10.1186/1471-2164-6-156

**Published:** 2005-11-08

**Authors:** Benoît Cadieux, Babykumari P Chitramuthu, David Baranowski, Hugh PJ Bennett

**Affiliations:** 1 Endocrine Laboratory, Royal Victoria Hospital, McGill University Health Centre, Montreal, Quebec, Canada; 2 Cancer Research Institute, UCSF, 2340 Sutter Street, N-231 San Francisco, CA 94143, USA; 3 Room L2.05, Royal Victoria Hospital, 687 Pine Avenue West, Montreal, Quebec, H3A 1A1, Canada

## Abstract

**Background:**

Progranulin is an epithelial tissue growth factor (also known as proepithelin, acrogranin and PC-cell-derived growth factor) that has been implicated in development, wound healing and in the progression of many cancers. The single mammalian progranulin gene encodes a glycoprotein precursor consisting of seven and one half tandemly repeated non-identical copies of the cystine-rich granulin motif. A genome-wide duplication event hypothesized to have occurred at the base of the teleost radiation predicts that mammalian progranulin may be represented by two co-orthologues in zebrafish.

**Results:**

The cDNAs encoding two zebrafish granulin precursors, progranulins-A and -B, were characterized and found to contain 10 and 9 copies of the granulin motif respectively. The cDNAs and genes encoding the two forms of granulin, progranulins-1 and -2, were also cloned and sequenced. Both latter peptides were found to be encoded by precursors with a simplified architecture consisting of one and one half copies of the granulin motif. A cDNA encoding a chimeric progranulin which likely arises through the mechanism of *trans*-splicing between *grn1 *and *grn2 *was also characterized. A non-coding RNA gene with antisense complementarity to both *grn1 *and *grn2 *was identified which may have functional implications with respect to gene dosage, as well as in restricting the formation of the chimeric form of progranulin. Chromosomal localization of the four progranulin (*grn*) genes reveals syntenic conservation for *grna *only, suggesting that it is the true orthologue of mammalian grn. RT-PCR and whole-mount *in situ *hybridization analysis of zebrafish grns during development reveals that combined expression of grna and grnb, but not grn1 and grn2, recapitulate many of the expression patterns observed for the murine counterpart. This includes maternal deposition, widespread central nervous system distribution and specific localization within the epithelial compartments of various organs.

**Conclusion:**

In support of the duplication-degeneration-complementation model of duplicate gene retention, partitioning of expression between grna and grnb was observed in the intermediate cell mass and yolk syncytial layer, respectively. Taken together these expression patterns suggest that the function of an ancestral grn gene has been devolved upon four paralogues in zebrafish.

## Background

In mammals, a single progranulin gene, also known as proepithelin, acrogranin and PC-cell-derived growth factor (PCDGF), encodes a glycoprotein precursor exhibiting pleiotropic tissue growth factor activity (reviewed in [1-4]). Progranulin is secreted in an intact form [5-8], or undergoes proteolysis leading to the release of its constituent peptides, the granulins [9-11]. Individual granulins have an approximate molecular weight of 6 kDa, and are structurally defined by the presence of 12 cysteines arranged in a characteristic motif: X_2–3_CX_5–6_CX_5_CCX_8_CCX_6_CCX_5_CCX_4_CX_5–6_CX_2 _[12]. Comparison of the biosynthetic origin of granulin peptides in various mammals reveals that all are commonly derived from a precursor composed of one amino-terminal half followed by seven non-identical copies of the granulin motif.

A role of progranulin in mammalian embryogenesis has been suggested. The exogenous addition of recombinant progranulin to eight-cell stage mouse embryos grown in culture *ex vivo *accelerates the onset of cavitation, stimulates the rate of blastocoel expansion, and leads to an increase in the number of trophectoderm cells compared to controls [13]. Conversely, the use of a progranulin function-blocking antibody arrests growth and prohibits embryo implantation [13,14]. These results are consistent with the growth-promoting activity of progranulin upon epithelial cells *in vitro*.

Despite these advances, the evolutionary history and phylogenetic distribution of the progranulin gene outside the mammalian radiation remain largely unexplored. In order to shed light on this issue, and to establish a model for studying the functional contribution of progranulin to vertebrate development, we undertook the characterization of the biosynthetic origins of progranulins in the zebrafish. The widely documented evidence in favor of a pan-genomic duplication event at the base of the teleost radiation [15], commonly referred to as 3R, predicts that the single mammalian progranulin gene will likely be represented by two zebrafish co-orthologues [16,17]. Results reported here demonstrate that, in zebrafish, progranulins arise as members of an extended gene family represented by two distinct architectures, in excess of that predicted by 3R.

Comparative chromosomal mapping of the various progranulin genes (*grns*) was performed to assist in the establishment of an orthologous relationship to their mammalian counterpart and to provide a point of reference to discuss the evolutionary origins of the distinct progranulin architectures. In support of the duplication-degeneration-complementation (DDC) model [18] gene expression analysis of the zebrafish progranulins reveals spatio-temporal divergence among the different family members, possibly reflecting extensive functional devolution of an ancestral form. Also, the occurrence of natural antisense transcription to some grns suggests that gene dosage may have influenced the retention of extra grn paralogues in zebrafish.

## Results

### Evidence for a progranulin multigene family in teleosts

**Figure 1:**
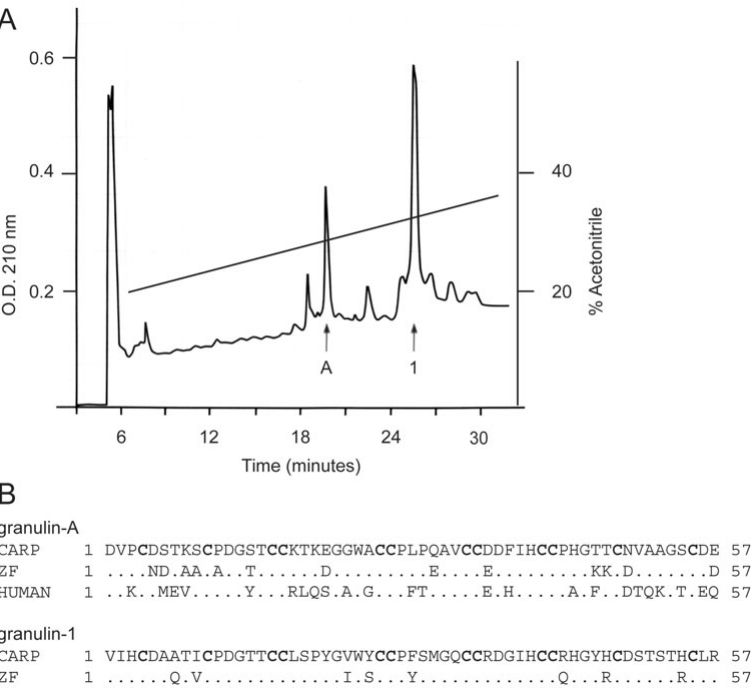
**Reversed-Phase HPLC purification of granulin-A and granulin-1 from extracts of two carp spleens**. Panel A: HPLC fractions derived from an extract of two carp spleens and enriched in granulin-1 immunoreactivity were further purified by HPLC using 0.13% heptafluorobutyric acid as counterion. Two major components were identified, a peptide sharing 58% sequence conservation with human granulin-A (peak A) and granulin-1 (peak 1), each indicated by arrows. Panel B: Sequence comparison of carp granulin peptides with their respective candidate orthologues deduced from cloned zebrafish cDNA sequences (this study), and human granulin-A. Numbers correspond to amino acid position. Characteristic cysteines are shown in bold.

A previous study indicated that major forms of granulin peptides found in hematopoietic organs of carp, *Cyprinus carpio*, differ in their relative abundance [19]. Specifically, extracts of carp spleen contained granulin-1 only, while the head kidney contained granulins-1, -2, and -3, arguing that some granulins found in teleost fish, unlike those found in mammals, have different biosynthetic origins [19]. We revisited the issue of the sole occurrence of granulin-1 in carp spleen and confirmed the earlier report. However, granulin-1 was found to co-purify with another member of the granulin family (Figure 1, Panel A). Sequencing of this peptide suggested that it is a close homologue of mammalian granulin-A, sharing 58% identity with the human peptide (Figure 1, Panel B). The isolation of a carp granulin peptide homologous to mammalian granulin-A suggested that teleosts synthesize a protein equivalent to mammalian progranulin. Carp is a tetraploid species known to express multiple copies of closely related genes that can complicate the study of the origins of multigene families [20]. Therefore, we chose to study the structure and expression of granulin genes in the zebrafish, a closely-related diploid teleost of the cyprinoforme order [21].

### Zebrafish progranulins are represented by two distinct architectures

#### Progranulin-1 and progranulin-2

**Figure 2:**
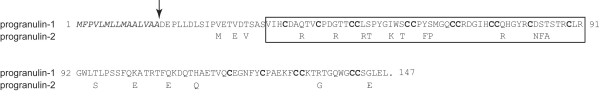
**Comparison of the deduced translated sequences for zebrafish progranulin-1 and progranulin-2**. Two zebrafish cDNAs sharing 92.3% identity (grn1 and grn2), each possessing a 441 nucleotide-long open reading frame (ORF), encode deduced precursors consisting of one full and one amino-terminal half granulin peptide, with a calculated mass of 14.5 kDa and 14.8 kDa, respectively. Both carry an identical signal peptide (italics), whose predicted cleavage site is indicated by an arrow. The predicted sequences for zebrafish granulins 1 and 2 are highlighted. Numbers correspond to amino acid position.

A PCR-based strategy using degenerate primers based on the amino acid sequence of carp granulin-1 led to the cloning of two zebrafish cDNAs sharing 92.3% identity and encoding structures homologous to carp granulin-1 and granulin-2, respectively (see Materials and Methods, Figure 2 and Additional File 1). In contrast with mammalian progranulins, the deduced architecture of these zebrafish precursors, named progranulin-1 (grn1) and progranulin-2 (grn2), consist of one full and one amino-terminal half granulin-like repeats only (Figure 2). Unlike progranulins A and B (see below), no potential N-glycosylation sites are found within the predicted progranulin-1 or progranulin-2 polypeptide structures. Subsequent cloning of the respective genes for *grn1 *and *grn2 *revealed that each is encoded on five exons (as shown for progranulin-1 in Additional Files 2 and 3), confirming that they are derived from distinct transcriptional units. The genomic sequences encoding *grn1 *and *grn2 *are interrupted by short introns in the exact equivalent positions to that observed within the mammalian *grn *gene [22,23]. Sequences at the splice junctions for the *grn1*, *grn2 *and *ASgrn1-2 *genes are depicted in Additional File 4.

#### Hybrid grn RNA

**Figure 3:**
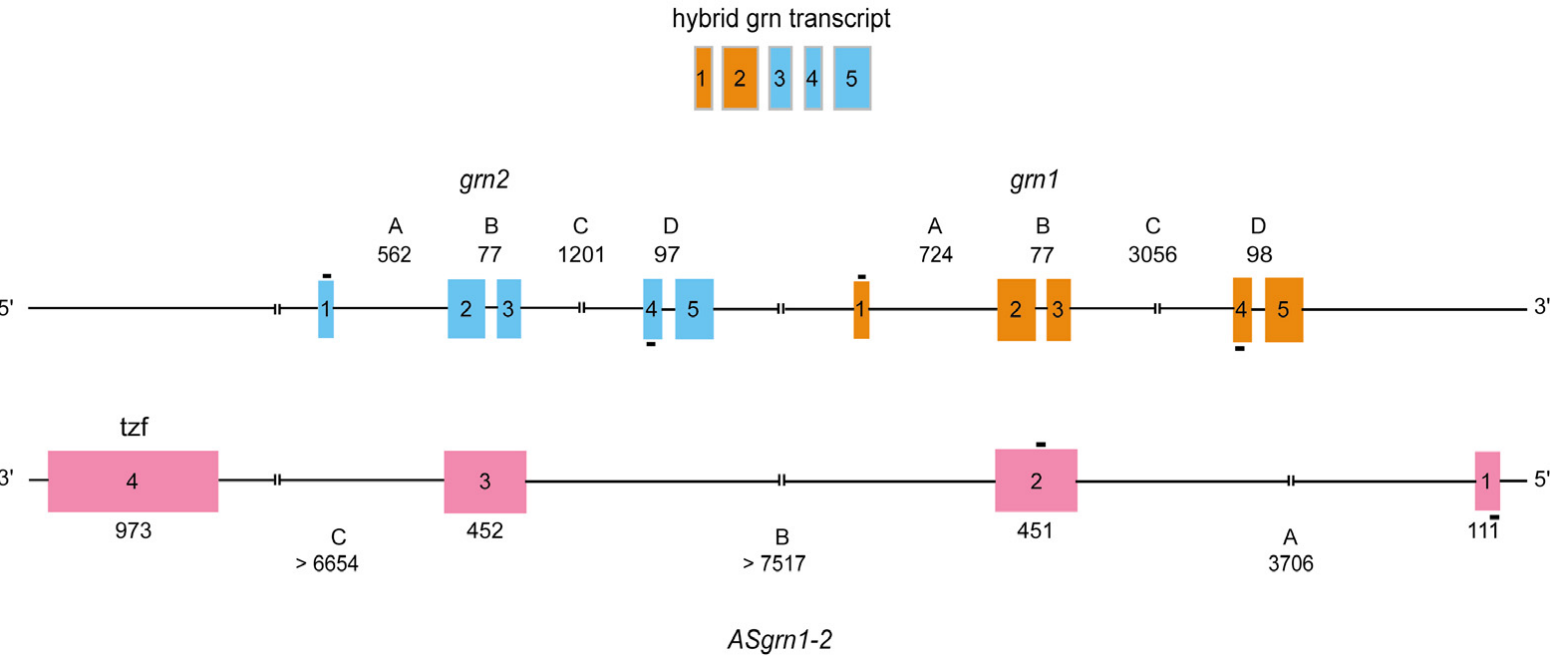
**Genomic organization of zebrafish grn1, grn2 and their complementary antisense gene**. Zebrafish grn1 and grn2 genes are found in tandem in a head-to-tail orientation and share an identical exonic organization (exons 1–5, orange and blue boxes, respectively), but differ in their respective intron lengths (A, C, D). The spliced and polyadenylated non-protein coding ASgrn1-2 is encoded on four exons (pink boxes) and shares exon/intron complementarity to both grn1 and grn2 (see text for details). Shown on top is a schematic representation of the chimeric progranulin transcript, suggesting trans-splicing as a mechanism for its generation. Bars (-) above or below exons indicate the relative position of primer pairs (grn1+2 forward and reverse; ASgrn1-2 forward and reverse) used for discriminating between ASgrn1-2 and combined grn1/grn2 expression using RT-PCR.

A cDNA encoding a hybrid progranulin identical in size to, and sharing 95.6% nucleotide sequence identity with both grn1 and grn2, was uncovered through our cloning strategy (see Materials and Methods). The nucleotide substitutions for hybrid progranulin are non-randomly distributed among the exons for *grn1 *and *grn2*, indicating that it is likely derived from the joining of the first two exons of *grn1 *with exons 3, 4 and 5 of *grn2 *(Figure 3 and Additional File 5). A hybrid of the opposite character was not detected (i.e. exons 1 and 2 of *grn2 *fused to exons 3, 4 and 5 of *grn1*). No evidence was found to suggest the existence of additional genomic sequences corresponding to *grn1 *or *grn2*, raising the possibility of a post-transcriptional mechanism underlying the origin of this chimeric structure.

#### Antisense progranulin1-2 gene

During the cloning of *grn1*, genomic sequences were used to perform BLAST searches for sequences deposited at NCBI [24]. An EST was detected (GenBank accession AW777232) whose sequence was an exact match to a portion of the *grn1 *gene, but in the reverse complement orientation. The full characterization of this EST, designated ASgrn1-2, revealed that it is spliced and shares exonic complementarity to exons 2 and 3, in addition to flanking intronic sequences, of both *grn1 *and *grn2 *genes (Figure 3 and Additional File 6). This observation suggested that *grn2 *is located upstream of *grn1 *in a head-to-tail organization, thus providing strong support for *trans*-splicing between the *grn1 *and *grn2 *primary transcripts as a mechanism for the genesis of the hybrid progranulin RNA (Figure 3).

In addition to its partial complementarity to *grn1 *and *grn2*, the last exon of the *ASgrn1-2 *gene (corresponding to nucleotides 1015 to 1989 of the cDNA) shares a high degree of sequence conservation with the tzf transposon [25], a subclass belonging to the *Tc1/mariner *superfamily of class II DNA mobile elements [25]. However, this mobile element is in the reverse complement orientation within the antisense transcript, and has undergone extensive mutations resulting from nucleotide insertions, deletions and point mutations (Additional File 7). Thus, *ASgrn1-2 *never possessed the ability to encode a translatable transposase protein, nor does it have a clearly predictable ORF in view of the presence of several termination codons in all three possible reading frames. For these reasons, this naturally occurring antisense transcript is considered to belong to the category of non-coding RNA genes.

#### The zebrafish co-orthologues of mammalian progranulin

**Figure 4:**
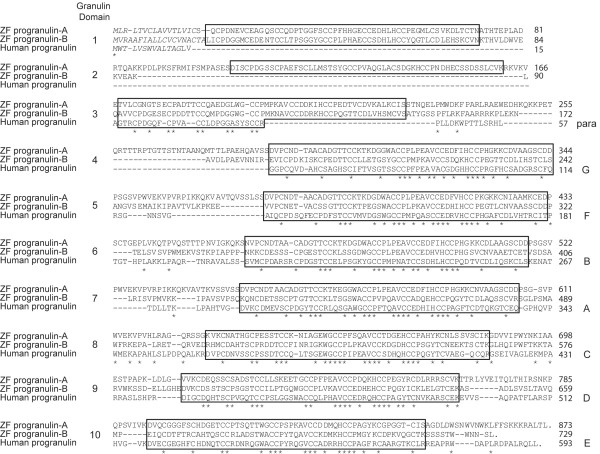
**Sequence comparison of zebrafish progranulin-A and -B with human progranulin**. Amino acid sequences were deduced from the cloned grna and grnb zebrafish cDNAs. Unlike human progranulin, which carries one-half and seven granulin peptide motifs, zebrafish progranulin-a and progranulin-b harbour 10 and 9 full copies of the granulin motif (boxed), and possess distinct putative signal peptides (italics). Sequences were aligned using the ClustalW method, and gaps were introduced as dashed lines for optimal alignment. Identical residues are indicated by an asterisk. Numbers on the right represent amino acid position. Human granulin motif nomenclature is listed (right).

Based on the finding of a carp granulin peptide whose sequence was homologous to mammalian forms of granulin (Figure 1, panel B), it was reasoned that these observations could be extended to the zebrafish. Using a strategy identical to that used for cloning a cDNA encoding granulin-1, we isolated a partial cDNA encoding a deduced zebrafish granulin-A peptide (data not shown). BLAST searches using this sequence retrieved two distinct ESTs from GenBank databases (accession AW174591 and AW184435), the former being identical to our cloned sequence. Sequencing of these cDNAs, referred to as progranulin-a (grna) and progranulin-b (grnb) respectively, revealed that each encodes a deduced granulins precursor bearing 10 and 9 tandemly repeated and non-identical granulin domains (Figure 4), demonstrating that the zebrafish progranulin repertoire is not limited to the simplified *grn *gene structures.

Zebrafish progranulin-A and progranulin-B are 48.6% identical over their aligned sequences, and each is similarly related to human progranulin with 44.8% and 42.9% identity, respectively. As expected, sequence conservation is seen primarily within the aligned granulin domains of the zebrafish precursors (Figure 4). However, the deduced granulins within the zebrafish grnA and grnB precursors cannot be aligned strictly on the basis of the mammalian nomenclature (i.e. in the order granulin-G, -F, -B, -A, -C, -D, -E) [5,26-28]. Indeed, progranulin-A contains five granulin peptides (domains 4, 6, 7, 8, 9) whose sequences bear close sequence similarity with one-another and with that of human granulin-A. In addition, zebrafish progranulin-A encodes a deduced granulin structure (domain 2) displaying the modified cysteine motif found in human granulin G, which is characterized by the absence of cysteine residues 4 and 7 (Figure 4).

#### Northern blot analysis

The size of the cloned sense and antisense zebrafish progranulin transcripts was assessed by northern blot analysis (Additional File 8). With the exception of the putative *ASgrna *(see below), the observed transcript sizes were in agreement with those predicted from cloned sequences. However, many pre-mRNA transcripts of higher than expected molecular weight were identified for several family members. Only grnb demonstrated the presence of splice variants of unknown composition.

#### Chromosomal mapping of zebrafish grns

**Figure 5:**
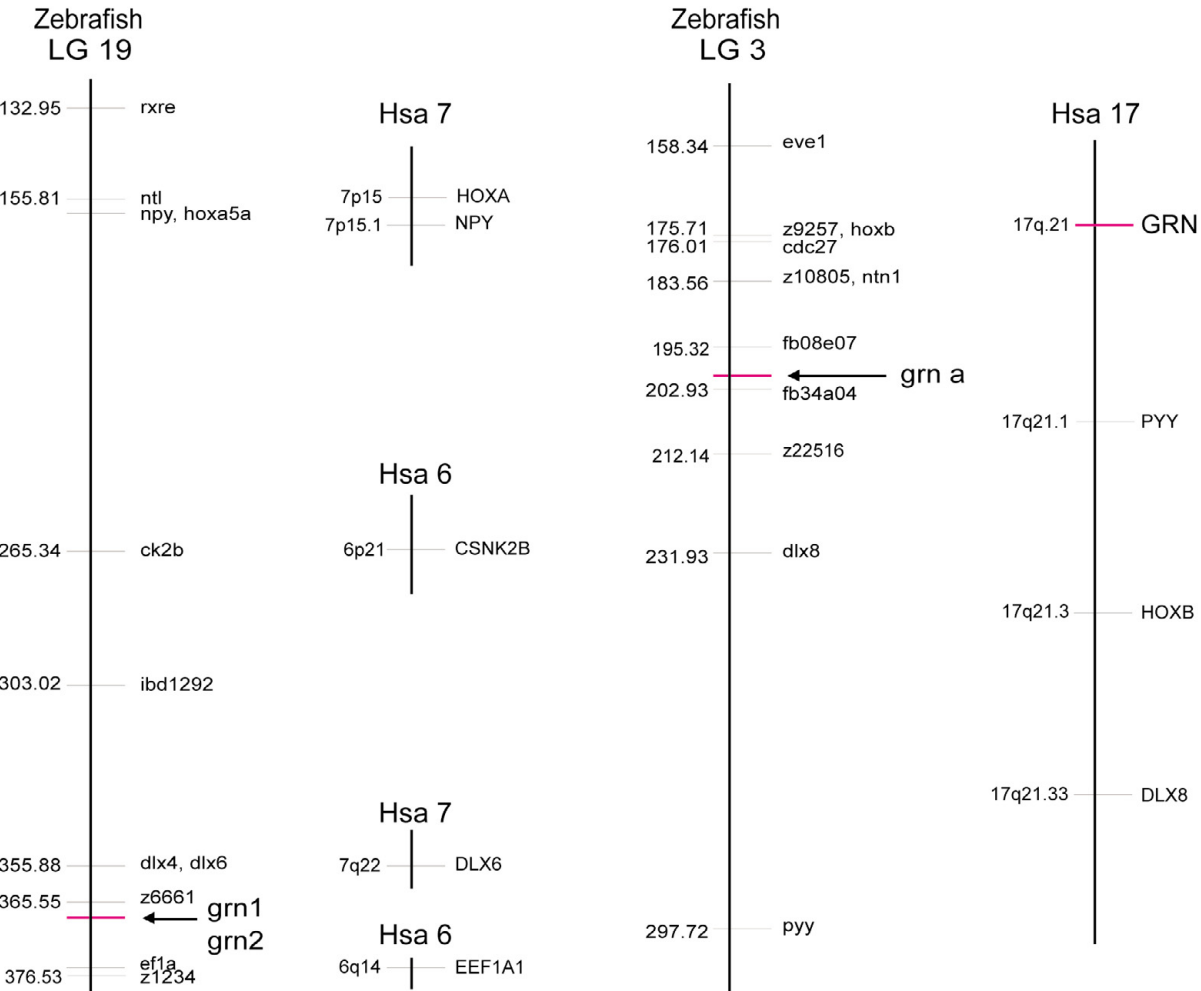
**Chromosomal assignment of zebrafish *grn *genes**. Zebrafish *grna *is located close to genes (*HoxB *cluster, *dlx8*, *pyy*) that form an extensive bloc of conserved synteny with human chromosome 17 (Hsa17), indicating an orthologous relationship to human progranulin (*grn*). Zebrafish *grnb*, in contrast, maps to LG24 in a region devoid of syntenic correspondence to zebrafish LG3 or Hsa17 (data not shown). *Grn1 *and *grn2 *map to LG19, in a region that finds scattered synteny to two human chromosomes (Hsa 6 and Hsa 7). The presence of *grn1 *and *grn2 *on a zebrafish chromosome bearing the *HoxA *cluster, *npy *and *dlx6 *genes (i.e. paralogues of genes linked to zebrafish *grna *and human *grn*), suggests that grn1 or grn2 may have originated in concert with the mechanism leading to emergence of duplicated *Hox *clusters at the base of the vertebrate radiation. Map position on zebrafish chromosomes (LG) is presented in centiRays where 1 centiRay = 148 kilobases, the estimated average breakpoint frequency for the LN54 RH panel.

The chromosomal localization of each *grn *gene was determined using the LN54 radiation-reduced mapping panel [29]. Primers are listed in Additional File 9. *grn1 *and *grn2 *are closely linked on linkage group (LG) 19, 5.98 centiRays (cR) from EST clone fb47h01 in the vicinity of the simple sequence length polymorphism (SSLP) marker z6661 consistent with their physical proximity deduced from the cloning of a chimeric transcript (Figure 5). *Grna *is located on LG3, 9.92 cR from SSLP marker z22516, in a region showing a clear syntenic correspondence with the chromosomal position of murine and human *grn *(Figure 5). In contrast, *grnb *maps next to SSLP marker z9325, and is 9.76 cR from EST fc18g06, located on LG24 (data not shown), and is not part of a known block of conserved synteny with zebrafish LG3 or human chromosome 17.

### Assessment of zebrafish *grn *gene expression by RT-PCR

#### Zebrafish grn gene expression in adult tissues

**Figure 6:**
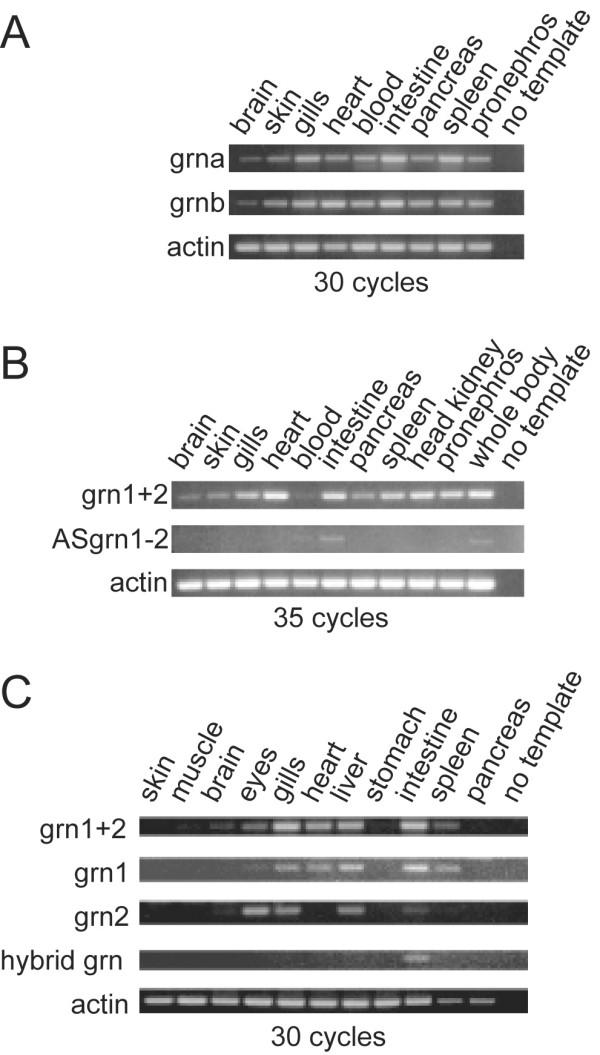
**RT-PCR Analysis of zebrafish grns in adult tissues**. *Panel A*: Zebrafish grna and grnb are ubiquitously expressed in various organs. *Panel B*: A comparison of the combined expression of grn1 and grn2 (grn1+2) relative to their antisense transcript. An increased number of cycles was used in the PCR to allow for the detection of ASgrn1-2 transcripts. *Panel C*: grn1, grn2 and hybrid grn are differentially regulated with the latter expressed only in the intestine. Results shown were generated using the forward and reverse1 primer pair (Additional File 10). Identical results were obtained using the forward and reverse2 primer pair (not shown). Hybrid grn was amplified using a grn1 forward and grn2 reverse primer combination. No product was obtained using a grn2 forward and grn1 reverse primer pair (not shown). Number of cycles for each reaction is indicated. Amplified PCR products were analyzed by electrophoresis next to a 100-bp DNA ladder. No template and amplification of actin mRNA were used as negative and positive controls, respectively. Similar results were obtained with two other experiments.

Semi-quantitative RT-PCR analyses were performed to examine the relative expression of the individual members of the zebrafish *grn *gene family in adult tissues. A list of primers used and size of the respective amplicons is listed in Additional File 10. Both grna and grnb are expressed in all adult tissues examined, including the gills, heart, multiple visceral organs, and at modest levels in the brain (Figure 6, panel A). Comparison of the expression of the smaller zebrafish paralogues (grn1 and grn2) relative to their mutual antisense gene in selected adult zebrafish organs (Figure 6, panel B), showed that the combined expression of grn1 and grn2 was qualitatively similar to that of grna and grnb (Figure 6, panels B and C). In contrast to the widespread combined expression of grn1 and grn2, low levels of ASgrn1-2 transcripts are detected in the blood and intestine (Figure 6, panel B). In agreement with the observed carp peptide expression profiles [19], the zebrafish spleen expresses grn1 only (Figure 6, panel C). In tissues that display overlapping expression, grn1 is the predominant form in the heart and intestine, while the eyes express higher levels of grn2 (Figure 6, panel C). Surprisingly, low abundance of the hybrid grn transcript, the authenticity of which was confirmed by sequencing of the amplicon, is detected exclusively in the intestine (Figure 6, panel C).

#### Developmental expression of Zebrafish grn genes

**Figure 7:**
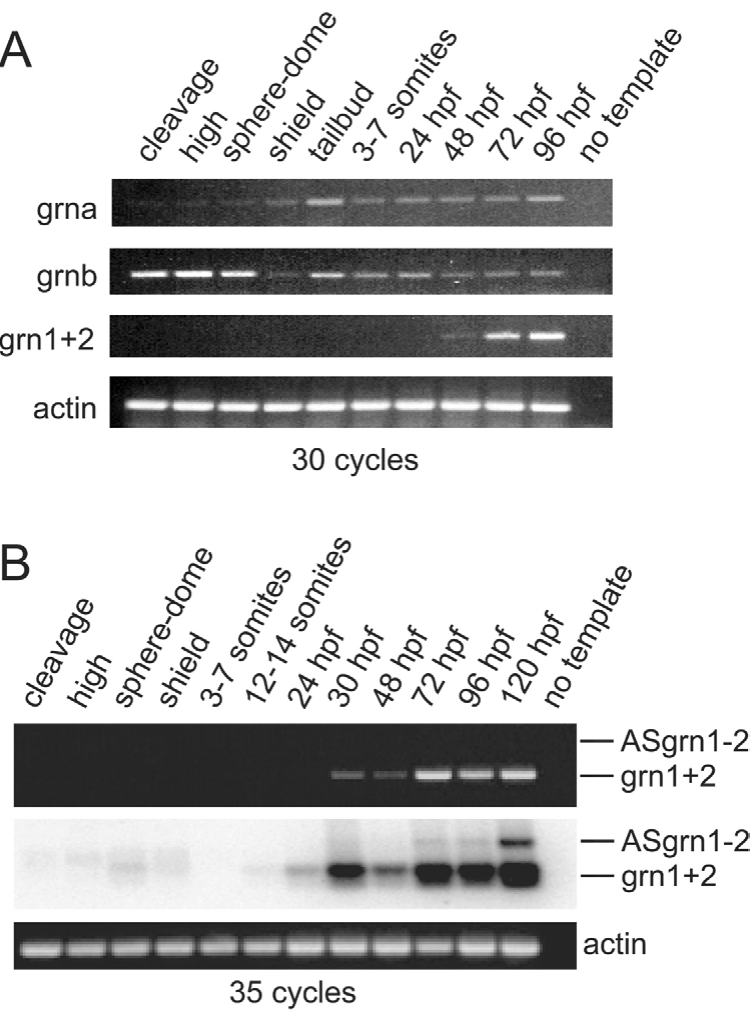
**RT-PCR analysis of zebrafish grn expression during development**. *Panel A*: grna and grnb transcripts are detected throughout all stages of development, whereas grn1 and grn2 expression is first detected by 48 hours post-fertilization. Maternal expression of grnb is more abundant than grna. *Panel B*: Combined expression of grn1 and grn2 relative to their antisense transcript. Ethidium bromide stain reveals the presence of sense transcription only (top). Detection of the antisense transcript is revealed by using a ^32^P-labelled oligo as probe that recognizes both sense and antisense amplicons after Southern transfer. Note weak expression of grn1 and/or grn2 at earlier stages of development. Numbers of cycles used for the PCR are indicated. No template and actin were used as negative and positive controls, respectively. Developmental stages are as follows according to Kimmel et al. 1995: cleavage (16-cell); high (mid-blastula, 3 hpf); sphere-dome (late blastula, 4–4.3 hpf); shield (50% epiboly, 6 hpf); tailbud (10 hpf); 3–7 somites (11–12 hpf); 12–14 somites (14–16 hpf); early (24 hpf) and late (48 hpf) pharyngula; hatching (72 hpf); ealy larval period (96 and 120 hpf). Gene specific primers and amplicon sizes are listed in Additional File 10.

Since divergence in expression patterns between duplicated genes is suspected to promote their retention [18], the temporal regulation of *grn *gene expression between the two *grn *gene classes during development was investigated by RT-PCR. Transcripts for both grna and grnb are maternally provided although grnb is more abundant (Figure 7, panel A). This trend continues following commencement of zygotic transcription until mid-epiboly (shield stage) where both transcripts are present at similar levels (Figure 7, panel A). In contrast, combined grn1 and grn2 expression is first noticeable during the late pharyngula period by 48 hours post-fertilization (hpf) (Figure 7, panel A). When using an increased number of cycling, the combined expression of grn1 and grn2 is detected as early as 30 hpf, while antisense transcript levels remain too low for detection using conventional ethidium bromide staining (Figure 7, panel B). Southern blot analysis demonstrates that antisense transcription occurs, albeit weakly, at 72 hpf and becomes more evident by 120 hpf (Figure 7, panel B).

### Assessment of zebrafish *grn *gene expression by whole mount *in situ *hybridization

In order to evaluate the relationship between sense and antisense transcription, and to gain insights into the potential contributions of the four grn paralogues to development, the spatio-temporal expression of zebrafish grns was monitored by whole-mount *in situ *hybridization. For all stages examined, sense and antisense riboprobes for *sonic hedgehog *were used as experimental controls since the tissue expression of this gene is discrete and well documented [30]. No non-specific hybridization signal for *sonic hedgehog *was detected (data not shown). Unless stated otherwise, the respective sense riboprobes to grna and grnb did not give rise to detectable signals.

#### Grna and grnb

**Figure 8:**
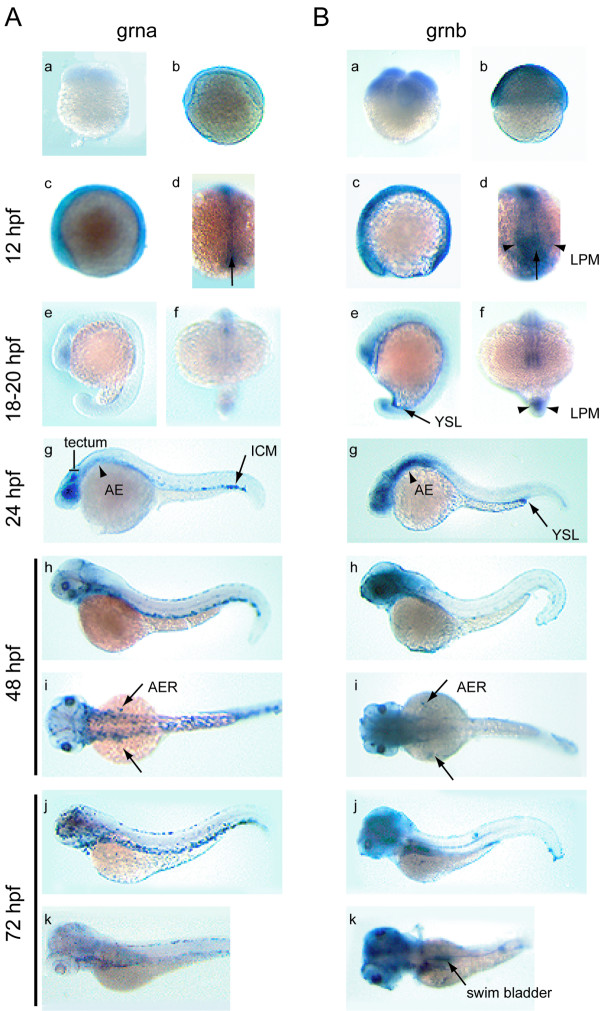
**Developmental expression analysis of zebrafish grna and grnb mRNAs by whole mount *in situ *hybridization**. An ontogeny of expression conducted for grna (A) and grnb (B) revealed similar expression patterns for these genes from fertilization to late segmentation stage (a–f), with grna being weaker than grnb. At the 4-cell stage (a) and 50% epiboly (b) ubiquitous expression is observed for grnb only. A lateral view of the 6-somite stage embryo (c) reveals discernable ubiquitous expression above background levels for grna, and increased grnb expression in the epithelium of the eye primordium and CNS, as well as the caudal region. In a dorsal view of the same animals (d), caudal expression in the axial mesoderm (arrow) is observed for both genes, whereas only grnb is detected in the paraxial mesoderm (arrowheads). Lateral (e) and frontal (f) views of the late somitogenesis stage embryo (18–20 hpf) show continued expression in the eye primordium, CNS and tailbud for both genes. In addition, grnb can be detected in the YSL (arrow) (e) and the adaxial cells (arrowheads) (f) flanking the axial mesoderm. At 24 hpf (g), grna expression is found in the tectum and eye retina, in a diffuse pattern in the anterior endoderm (arrowhead) and in a punctuate pattern within the ventral tail region of the ICM (arrow), whereas elevated expression persists for grnb in the forebrain, midbrain and ventral hindbrain region, the eyes, as well as in the YSL, concentrated at the tip of the yolk extension (arrow). In a lateral view at 48 hpf (h), grna expression in the ICM extends rostrally, is detected in the head vasculature, and is now apparent in the skin epithelium, whereas in a lateral view (i) both grna and grnb are transiently expressed in the AER of the pectoral fin buds (arrows). At 72 hpf (j), grna, but not grnb, is expressed in presumed dispersed leukocytes, while in a dorsolateral view (k), grnb can be detected in the swim bladder (arrow). AE, anterior endoderm; AER, apical ectodermal ridge of the pectoral fin buds; ICM, intermediate cell mass; LPM. lateral plate mesoderm; YSL, yolk syncytial layer.

**Figure 9:**
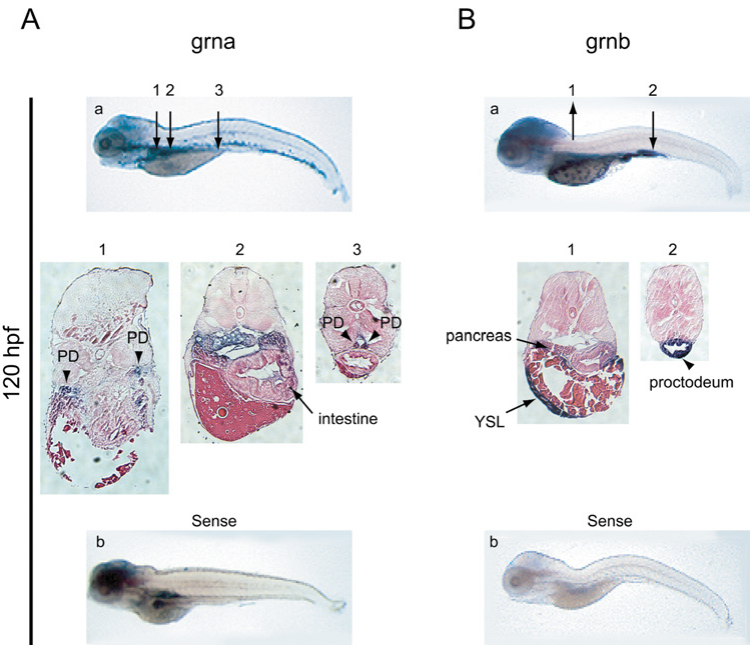
**Expression analysis of zebrafish grna and grnb mRNAs by whole mount *in situ *hybridisation at 5 dpf**. At 120 hpf grna (A) exhibits widespread expression in the visceral region, including the pronephric kidneys (arrowheads in sections 1 and 3) and intestine (arrow in section 2), while grnb (B) remains expressed in the YSL and pancreas (arrows in section 1) and is strong in the proctodeum region of the intestine (arrow in section 2). At this stage, a hybridization signal for the sense riboprobe to grna, but not grnb, is detected in the brain, intestine and pronephric ducts (b). Numbered arrows denote the position of corresponding sections shown below (magnified 10×). PD, pronephric ducts; YSL, yolk syncytial layer.

The low abundance of transcripts for grna relative to grnb revealed by RT-PCR during early embryogenesis (Figure 7, panel A) is reflected in the relative signal intensity for these transcripts by whole-mount *in situ *hybridization from the 4-cell to late segmentation stage (18–20 hpf) (Figure 8, panel A and B, a–f). Weak, ubiquitous grna expression can first be detected by 12 hpf (6 somite stage), with slightly stronger signal in the hypoblast (Figure 8, panel A, c, d). Grnb expression remains similarly ubiquitous, however defined regions of heightened expression are evident including in the hypoblast, central nervous system (CNS), optic epithelial layers, ear primordium, lateral plate mesoderm (LPM) and tailbud (Figure 8, panel B, c, d and data not shown). As the embryo matures to late segmentation stages (18–20 hpf), low levels of grna expression become confined to the eyes, tectum and tailbud (Figure 8, panel A, e, f), whereas grnb expression undergoes further regionalization within the eyes and CNS, the caudal region of the notochord and surrounding adaxial cells, and in the yolk syncytial layer (YSL) (Figure 8, panel B, e, f). During the pharyngula period (24–48 hpf) and hatching stage (72 hpf), overlapping expression patterns between grna and grnb include the pharyngeal and anterior visceral endoderm, the skin epidermis (Figure 8, panels A and B, g–i), and the pronephric tubules, albeit at modest levels (data not shown). In addition, both genes are expressed temporally within the apical ectodermal ridge (AER) between 36 and 72 hpf (Figure 8, panels A and B, h, i and data not shown). Divergent expression of the zebrafish co-orthologues of mammalian progranulin is also notable during this period (24–72 hpf). For instance, only grna is strongly expressed in the intermediate cell mass (ICM) caudal region, the lens and retina as well as the tectum of the 24 hpf embryo (Figure 8, panel A, g). Grna is also uniquely expressed at low levels within the forming head vasculature and aorta at 48 hpf (Figure 8, panel A, h–i and data not shown), and exhibits relatively increased leukocytic expression from 48 to 72 hpf (Figure 8, panel A, h–k). In contrast to that observed for grna, grnb is expressed within the YSL and often found concentrated at the end of the yolk extension. Sustained high levels of grnb were observed within the brain at all stages examined (Figure 8, panel B, g–k) and in the swim bladder by 72 hpf (Figure 8, panel B, k). Patterns observed during the hatching period generally persist and are accentuated in the 5 day-old larva (Figure 9, panels A and B). In addition, grna can be detected in the epithelial lining of various visceral organs, in particular the pharynx, intestine, swim bladder and pronephric ducts (Figure 9, panel A, a). Weak staining in the dorsal aorta but not the posterior cardinal vein can sometimes be noticed in whole-mount and through sectioning of the animal (Figure 9, panel A, a). Likewise, grnb is widely expressed in the visceral region of the larval stage animal, in particular the intestine, pancreas and YSL (Figure 8 and 9). Weak expression is also detected for both genes in the olfactory epithelium and in the presumptive thymus as bilateral patches located caudal to the eyes (data not shown).

The sense riboprobe corresponding to grna but not grnb detects staining in the brain, intestine and pronephros at this stage (Figure 9, panel A and B, b), suggesting either non-specific hybridization or the presence of an antisense transcript. This observation prompted a search for sequences deposited at GenBank corresponding to parts of the grna cDNA sequence, but in the reverse complement orientation. Three unidirectionally cloned ESTs (accessions CD585878, CD585963 and CD596001) were all revealed upon sequencing to correspond to a 914 nucleotide long cDNA sharing perfect complementarity to nucleotides 2701–3614 of the grna cDNA (Additional File 11, panel A). This sequence corresponds to the 3'UTR region of the *grna *gene and is not bisected by an intron at the genomic level. This precludes the conclusion that this candidate antisense transcript is not an artifact of cloning. To confirm its directionality, cDNA was synthesized for subsequent PCR amplification using a primer located within the 3'UTR exon of grna that shared complementarity to ASgrna (sense relative to grna) or using another primer that was located downstream of the cloned ASgrna sequence and within a known intron for *grna *(Additional File 11, panel A). This RT-PCR strategy suggests that the ASgrna deduced from cloned EST sequences may represent a splice variant, and that antisense transcription extends further in the 3'direction (Additional File 11, panel B).

#### Grn1 and grn2

**Figure 10:**
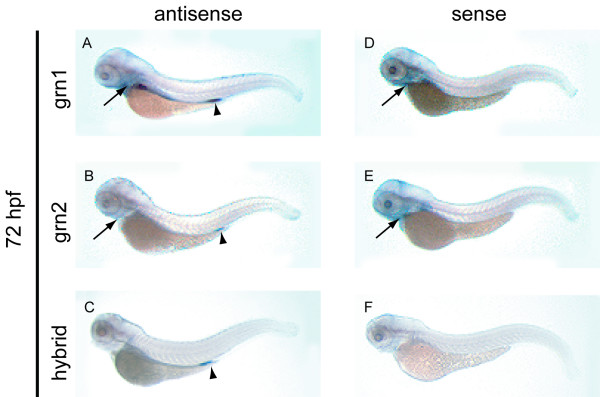
**Expression analysis of grn1, grn2, hybrid grn, and ASgrn1-2 in the hatching stage zebrafish embryo by whole mount *in situ *hybridization**. *Panel A*: grn1 is expressed in the intestine and pharyngeal region (arrow), and at low levels in the pronephric ducts. *Panel B*: In contrast, grn2 mRNA is only weakly detected in the pharyngeal region (arrow) and the proctodeum (arrowhead), and is occasionally found in dispersed leukocytes (not shown). *Panel C*: The abundance of the *trans*-spliced product (hybrid grn) is stronger than grn2 in the proctodeum (arrowhead), but absent in the pharyngeal region. *Panels D–F*: The corresponding sense riboprobes to grn1 and grn2, but not to hybrid grn, detect ASgrn1-2 expression in the pharyngeal region (arrows). These expression patterns were reproduced in at least three independent experiments.

As expected from the RT-PCR data (Figure 7, panel A), expression for grn1 or grn2 is not detected by *in situ *hybridization in early development (data not shown). Expression of grn1 is detected in the intestine and the pharyngeal region of the 3-day old animal (Figure 10, panel A) and at very low levels in the pronephros (data not shown). Grn2 does not share this expression pattern and is detected at low levels in the proctodeum (Figure 10, panel B) and is often detected in a few sporadic peripheral leukocytes (data not shown). In contrast hybrid grn is detected at relatively high levels in the proctodeum. (Figure 10, panel C). This specific expression pattern is confirmed by examining the signal obtained using sense riboprobes corresponding to grn1 and grn2 that, after prolonged exposure, detect weak signals from their complementary transcript (ASgrn1-2) in the pharyngeal region of these animals (Figure 10, panels D and E). Notably, the use of a sense riboprobe corresponding to hybrid grn (Figure 10, panel F) does not replicate the expression pattern observed for ASgrn1-2 (Figure 10, panel D and E).

**Figure 11:**
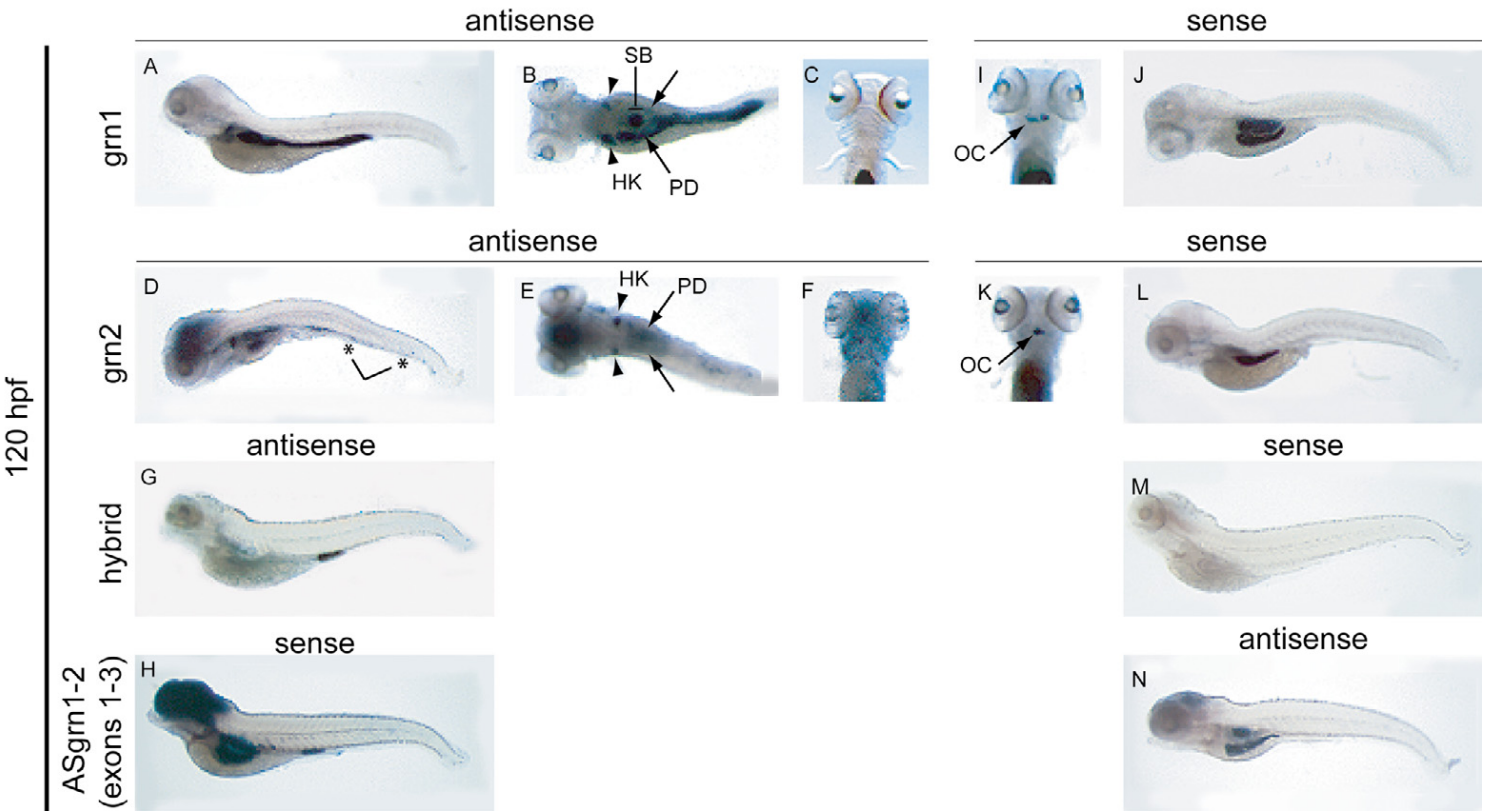
**Expression analysis of grn1, grn2, hybrid grn, and ASgrn1-2 in the 5 day-old zebrafish larva by mRNA in situ hybridization**. *Panels A–C*: grn1 is expressed in the intestine (arrow), swim bladder, and more abundantly in the head kidneys (arrowheads) than in the pronephric ducts. *Panels D–F*: grn2 is expressed similarly to grn1 in the head kidneys (arrowheads) and pronephric ducts, but is undetected in the intestine. In contrast, grn2 is strongly expressed in the brain and the branchial jaw region (compare A with D, and C with F), is distributed in a punctuate pattern along the ventral region of the animal in presumed myeloid progenitors (asterisks in D), and often found in randomly dispersed leukocytes (large cells in F). *Panel G*: Hybrid grn is found exclusively in the proctodeum. *Panel H*: The sense riboprobe to ASgrn1-2 (devoid of the tzf sequence) recapitulates the combined expression patterns for grn1 and grn2. *Panels (I–N)*: Sense riboprobes to grn1 (I,J) or grn2 (K,L), but not hybrid grn (M), show that antisense transcription occurs in the jaw region (arrows in I and K), the swim bladder, and in the mid-region of the intestine, in a pattern identical to that observed for the antisense probe corresponding to ASgrn1-2 (N). B, E: dorsal views; C, F, I, K: ventral views. For each target mRNA, the use of corresponding sense and antisense (AS) riboprobes is indicated. OC, presumed ossification center; HK, head kidney; PD, pronephric duct; SB, swim bladder.

In the 5 day-old larva, specific grn1 expression is observed in the intestine and the swim bladder, whereas low levels can be seen in the pharynges (Figure 11A–C). Elevated levels of expression for grn2 in the brain, the pharyngeal jaw region, and in presumed peripheral leukocytes (Figure 11D–F), contrast with the pattern observed for grn1. However, both genes share higher levels of expression in the anterior or head kidneys and pronephric ducts (Figure 11B and 11E). *In situ *hybridization of cross sections of the animals indicates that grn1 and grn2 expression, like that of grna and grnb, occurs in the epithelial lining of the visceral organs (data not shown). The chimeric transcript remained restricted to the proctodeum where it is expressed more abundantly than grn2, and was not detected in leukocytes at this stage (Figure 11G). In turn, the antisense gene to grn1 and grn2 is expressed in the brain, swim bladder, and the middle segment of the intestine (Figure 11H).

## Discussion

### Zebrafish progranulins: simplified molecular forms and orthologues of the mammalian gene

The granulin peptide family was originally discovered as a component of the granule fraction of mammalian phagocytic leukocytes. A series of related cysteine-rich peptides (designated granulins A, B, C and D) were purified from extracts of human neutrophils [9]. The definition of the structure of human progranulin as a glycoprotein bearing multiple copies of the granulin motif made it apparent that granulin peptides are generated through proteolytic cleavage of this precursor within the phagolysosomal compartment of the neutrophil. This explains the roughly equimolar ratios observed for members of the granulin family that are co-packaged within this subcellular compartment. The hematopoietic tissues (spleen and head kidney) of the carp (*Cyprinus carpio*) were also shown to be abundant sources of three granulin-like peptides (granulins-1, -2 and -3) [19]. However, the non-stoichiometric ratios observed for carp granulins-1,-2 and -3 suggested that the granulin gene family in this teleost species may expand beyond the prototypic single *grn *gene found in mammals. Specifically, carp spleen contains mainly granulin-1, whereas granulins-1, -2 and -3 were found in extracts of the head kidney (Belcourt et al. 1993). To simplify the identification of teleost granulin gene family members, the zebrafish (*Danio rerio*) was chosen based on its usefulness as a model of vertebrate development and disease.

The presence of extra gene paralogues in teleost fish has been extensively documented, often [15,31,32] but not invariably supporting [33,34] the hypothesis that the actinopterygian (ray-finned) lineage underwent an additional round of genome duplication (3R) subsequent to diverging from the sarcopterygian (lobe-finned) lineage approximately 450 mya. Specific examples include the *Hox *clusters [16,35], the annexins [36], the claudins [37] and the Nodal-related genes *squint *and *cyclops *[38]. Further, comparative chromosomal mapping studies have shown that duplicated zebrafish genes often reside on distinct chromosomes that exhibit extensive blocs of conserved synteny with their mammalian counterpart [39,40]. Using this approach, it has been estimated that approximately 20% of human genes may be represented by two co-orthologues in the genome of zebrafish [41].

As predicted by 3R, we demonstrate that *grns *are members of an extended gene family in zebrafish. We have identified two deduced precursors, progranulins A and B, harbouring 10 and 9 granulin peptide repeats respectively that bear close structural and sequence relationship to human progranulin (Figure 4). *Grna *was localized to a region of LG3 known to show syntenic correspondence to where human *grn *is found on human chromosome 17 (Figure 5). *Grnb *was localized to LG 24 rather than being positioned on LG12, predicted to bear synteny with LG3 [40]. Despite this apparent discrepancy, a co-orthologous relationship between *grna *and *grnb *relative to mammalian *grn *is supported by their sequence conservation as well as their extensive overlapping expression patterns observed during development.

Two smaller *grn *genes, *grn1 *and *grn2*, each encoding one full and one amino-terminal half copies only of the granulin motif, were also characterized (Figure 2). Interestingly, a common ancestry of the smaller zebrafish *grn *genes with *grna *and *grnb*, and thus mammalian *grn*, is implied by conservation of the strict exonic organization that these genes display. However, it is unlikely that *grn1 *and *grn2 *arose from the postulated whole genome duplication event corresponding to 3R since they were found to be localized in tandem on LG19 (Figure 5). It is notable that both *grn1 *and *grn2 *are linked to a *Hox *cluster and *dlx *gene paralogues, similar to that observed for *grna*. This suggests that a smaller *grn *may have originated coincidentally with the duplication of a Hox-bearing chromosome in a primordial species. Evidence suggests that this putative structure would then have been retained within the teleosts but lost within the sarcopterygian line of evolution leading to mammals.

### Analysis of progranulin expression by RT-PCR

The DDC model predicts that an important driving force behind the retention of duplicated genes is the devolution of an ancestral function onto the resultant pair through quantitative and qualitative changes in gene expression which, when combined, may reflect the sum of the ancestral expression pattern [18]. Thus, it was of interest to determine the extent of expression partitioning and overlap between the two *grn *gene classes during development by RT-PCR and to compare these patterns to those known for the mammalian counterpart.

As an initial survey of the differential expression patterns of the zebrafish *grns*, we conducted semi-quantitative RT-PCR analyses using adult tissues and staged embryos. Similar to the well documented widespread expression pattern of human [26,27,42], rat [28], mouse and guinea pig [5]*grns *in several tissues and cell lines of epithelial, mesenchymal, and hematopoietic origin, both grna and grnb were observed to be expressed ubiquitously in several adult zebrafish organs. In contrast, grn1 and grn2 exhibit a more restricted pattern of expression. It was previously shown that carp granulin-1 and granulin-2 peptides are differently distributed in the spleen and head kidneys of the carp [19]. Interestingly, RT-PCR experiments demonstrate that the expression of the homologous structures in zebrafish were similarly uncoupled at the level of mRNA, (Figure 6, panel C), reflected in a lack of detectable zebrafish grn2 expression in adult spleen. Also, grn1 appears to be the predominant form in the heart, while the eyes express higher levels of grn2. Whether the widespread pattern of expression for zebrafish grns is due in part to leukocyte entrapment in some organs, cells known to express granulins in carp [43] and goldfish [44] cannot be determined using this experimental approach.

Other notable differences in the expression of the paralogous and orthologous pairs of genes were observed. First, maternal transcripts for grna and grnb are readily detectable, with grnb showing higher abundance than grna until early somitogenesis when the two genes become expressed at similar levels (Figure 7, panel A). In contrast, although very low levels of grn1 mRNA are also detected in the newly-fertilized egg, the combined expression of grn1 and grn2 only becomes detectable by 30 hpf, at a stage when most organogenesis is well advanced (Figure 7, panel A). Thus, the absence of grn1 and grn2 expression prior to the onset of zygotic expression argues that these genes are functionally dispensable in early embryogenesis.

### Analysis of progranulin expression by whole mount *in situ *hybridization

#### Early expression

In order to determine possible roles for the grns during development, their spatio-temporal distribution was examined by whole mount *in situ *hybridization. Overall, grna and grnb expression patterns share conserved features with their murine orthologue in the early embryo. Transcripts for both zebrafish co-orthologues are maternally deposited and remain ubiquitously expressed subsequent to the onset of zygotic transcription (mid-blastula transition – 3 hpf). Similarly, in a pattern that reflects the replacement of maternal mRNAs with zygotically expressed transcripts [45], murine grn mRNA levels fall rapidly after egg fertilization, reaching negligible levels as early as the 2-cell stage, but rise again to detectable levels by the eight-cell stage [13] Notably, this precedes the morula stage and subsequent blastocyst stage when the epithelium is first formed. It is interesting to note that during zebrafish epiboly, which comprises the morphogenetic movements of the blastoderm towards the vegetal pole (dome to tailbud stage – 4.5 hpf to 10 hpf), grna and grnb are still ubiquitous but more intense in the outer enveloping monolayer of cells (EVL), which ultimately will give rise to an epithelium covering the blastoderm. Expression of these grns in the EVL and later in the skin ectoderm is reminiscent of the more elevated levels of expression for grn in the apical surface of the mouse blastocyst epithelium, the trophectoderm, relative to the inner cell mass population [13].

#### CNS

Although several regions display increased expression of grna and grnb during brain segmentation (Figure 8, panel A and B, c–f), regional specificity is more apparent at 24 hpf. Distinct or non-overlapping patterns observed include grna expression within the tectum and a more expansive grnb expression pattern encompassing the midbrain-hindbrain boundary, tegmentum and telencephalon (Figure 8, panel B, g). Despite the expression of both grna and grnb in the epithelial lining of the eyes and lens, transient expression within the retina and tectum is noticeable for grna only, suggesting that this orthologue may affect the development of the retinotectal projections. Similar functions may also be implied for mammalian grn, given its expression within the retinal glia during murine development [46]. Interestingly, murine grn expression is abundant throughout the central and peripheral nervous system, similar to that observed for grnb at later developmental time points within the zebrafish CNS (Figure 8 and 9). The generally unrestricted gene expression pattern suggests a role for progranulin in cell survival or proliferation or as a competence factor. Indeed, mammalian grn has been demonstrated to be a potent glial cell mitogen *in vitro *[47] and is consistently up-regulated in malignant human gliomas [47,48]. Grn2 and ASgrn1-2 but not grn1 demonstrate similar unrestricted expression within the zebrafish brain (Figure 11d,h). Taken together these expression patterns suggest that CNS development in the zebrafish may involve a functional interplay between the various molecular forms of granulin.

The only known functional and physiological contribution that *grn *gene expression is known to make during neural maturation is its involvement in the sexual differentiation of the male rat brain. It has been shown that male sexual behaviour is associated with steroid-dependent *grn *expression in the male neonatal hypothalamus [49,50]. Similar distinctions in *grn *gene expression were not determined in the present study given that sexual differentiation of zebrafish gonads occurs much later and spans roughly 21–28 dpf [51].

#### Endoderm

The expression of the zebrafish *grn *gene family members also displays overlapping and distinctive patterns with respect to the endoderm and tissues derived from this germ layer. At 24 hpf both grna and grnb can be found within the pharyngeal and foregut endoderm, whereas only the latter is located within the YSL (Figure 8, panels A and B, g). Both transcripts maintain a degree of dispersed endodermal expression until 120 hpf where grna is located within the epithelial lining of the stomach and anterior intestine while grnb can be found within the YSL, pancreas and proctodeum (Figure 8 and 9).

Unlike the expression of grna and grnb, the paralogues grn1, grn2 and hybrid granulin are highly abundant and for the most part restricted to pharyngeal and visceral endodermal derivatives from 72 hpf onward. Although grn1 and grn2 demonstrate some endodermal tissue-specific expression, these transcripts often co-localize (Figure 11a,b,d,e). The restricted expression of hybrid granulin within the proctodeum is particularly striking (Figure 11g). Consistent with the manner in which the hybrid transcript is formed, both grn1 and grn2 are likewise expressed in the proctodeum. However, expression of these two transcripts overlaps within a large portion of the intestine and stomach where no hybrid grn transcript is found. This suggests that wherever the hybrid grn is observed, the generation of this chimeric peptide must be a highly regulated process and that a specific function is implied.

In contrast to the observed grn distribution in zebrafish, murine grn is not detected within developing endodermal derivatives, with the exception of adult deep crypt enterocytes [42,46]. This suggests that mammalian dependence on grn expression may be developmentally restricted to endodermal-epithelial transitions in the gut or subsequent maintenance of this organ. Alternatively, it is possible that endodermal expression of zebrafish grns reflects a species-specific requirement.

#### Hematopoietic tissue

Of particular interest in regards to functional equivalence between species and functional separation between duplicated co-orthologues, is the almost complete partitioning of grn hematopoietic expression onto grna and to a much lesser extent grn2. In zebrafish, primitive hematopoiesis occurs within the anterior ICM from where nucleated erythroblasts originate and myeloid cells that can be seen circulating at 24 hpf [52]. Hematopoietic stem cells (HSCs) are then believed to populate the dorsal aorta and yolk sac which represent the zebrafish equivalent of the mammalian aorta-gonad-mesonephros, the tissue presumed to be responsible for later definitive erythropoiesis [53,54]. The posterior ICM, located within the ventral tail and positive for molecular markers of all three hematopoietic lineages, may represent a secondary zebrafish HSC population or region required for HSC maturation [55]. Unlike the murine model, zebrafish definitive hematopoiesis undergoes a migratory transition from the dorsal aorta/ventral tail to the kidney (roughly 96 hpf), without the involvement of liver or bone marrow.

In accordance with expression patterns mentioned previously, grna only acquires distinctive tissue-specificity at 24 hpf, as is the case for its prevalence within the caudal ICM (Figure 8, panel A, g–j), restricting its involvement to definitive hematopoietic waves. Grna can be found at low levels within the dorsal aorta at 48 hpf (data not shown) and is highly expressed within the caudal-ventral tail region throughout all stages post-24 hpf (Figure 8 and 9), suggesting its involvement in multiple hematopoietic lineages. Significantly, sustained *grna *expression in this hematopoietic organ is coupled with the appearance of *grna*-expressing leukocytes dispersed throughout the animal with levels that peak at approximately 72 hpf (Figure 8, panel A, j–k). Thus *grna *expression in presumed granulocytes all over the body of the animal may suggest its involvement in the innate immune response of the host. Grn2 expression is also found within peripheral leukocytes, but in a sporadic pattern that is distinct from that observed for grna. Whether these differences reflect leukocyte sub-populations or activation states for these cells has not been addressed. Commensurate with the transition of ICM to kidney as the major site of hematopoiesis, grna can be found within the pronephric ducts (Figure 9) along with grn1 and grn2, which are also present in the head kidney (Figure 11, panel B and E).

Mammalian grn exhibits a similar expression pattern, particularly in neutrophils. Furthermore, murine grn can modulate the inflammatory response during wound healing, acting as both a chemokinetic factor and inhibitor of neutrophil degranulation and respiratory burst [56,57]. It remains to be determined whether grna supports a similar role in zebrafish.

### Chimeric transcription

During the initial degenerate primer amplification of cDNAs encoding grn1 and 2, a third cDNA was cloned and identified as sharing strict identity with portions of both grn1 and 2. Interestingly, this granulin-hybrid showed 100% identity with exons 1 and 2 of *grn-1*, and with exons 3, 4 and 5 of *grn-2*, suggesting that this hybrid cDNA may represent a splicing of granulin-1 and 2 primary transcripts (Figure 3). Chimeric transcripts usually result from one of the following mechanisms: chromosomal translocations, transcription of neighboring genes as a single transcription unit or alternative splicing in *trans*. In all cases, joining of exons is predicted to occur through the recognition of canonical splice acceptor and donor sites.

A hybrid granulin structure has been previously reported through cloning of cDNA sequences in the rat [27]. Specifically, a structural splice variant of progranulin cDNA was retrieved and predicted to encode a granulin domain consisting of the amino-terminal domain of granulin-C fused to the carboxyl-terminal domain of granulin-D, consistent with the removal of an exon from the larger primary transcript [22,28]. The zebrafish hybrid grn described here likely originates through a mechanism other than alternative splicing from a larger primary transcript since the *grn2 *gene is located 5' to the *grn1 *gene (Figure 3). This topology was confirmed through the cloning and structural analysis of the partially complementary *ASgrn1-2 *gene. Also, we found no evidence for the presence of additional grn1-like genomic sequences located upstream of the *grn2 *gene, or elsewhere in the genome by Southern analysis (data not shown). In particular, no equivalent of carp grn3 was found in zebrafish. These observations suggest that the presence of hybrid grn in zebrafish likely occurs through a splicing reaction in *trans *between grn1 and grn2 pre-mRNAs, similar to the mechanism originally documented in trypanosomatids [58]. Although rare, scrambled or intergenic RNA molecules consisting of exons originating from distinct genes through a *trans*-splicing reaction have also been documented in vertebrates. For instance, acyl-CoA:cholesterol acyltransferase-1 (*ACAT-1*) and the CYP3A family of P450 cytochrome genes produce hybrid mRNA variants in humans [59,60]. *Trans*-splicing of the voltage-gated sodium channel in response to nerve growth factor stimulation, further suggests that this alternative mode of splicing can be a regulated process [61]. Indeed, there is evidence suggesting that splicing in *trans *may be facilitated through the recognition of regulatory elements within transcript sequences called splicing enhancers that require binding of SR proteins for activity [62]. Whether grn1 or grn2 harbour a necessary enhancer sequence that could explain the directionality of hybrid grn is currently not known. We believe the hybrid granulin represents the first example of *trans*-splicing with regards to the modification of a growth factor gene product.

### Antisense transcription

Although the majority of deposited zebrafish EST library sequencing confirmed the existence of grn1 and grn2, one particular sequence (AW777232) corresponded to the exact reverse complement to both *grn1 *and *grn2 *within the same exon/intron spanning region (exons 2–3 and intervening intron), and was named *ASgrn1-2 *accordingly. In addition, *ASgrn1-2 *harbours sequences for an extensively mutated *tzf *transposon (Tc1/mariner superfamily) in its last exon, but in the reverse complement orientation (Figure 3 and Additional File 7). Despite its polyadenylation, the lack of an ORF classifies ASgrn1-2 as a non-coding RNA. To our knowledge, ASgrn1-2 represents the first example of a single spliced transcript with antisense complementarity to two tandemly organized paralogous protein-coding genes.

The existence of ASgrn1-2 has potential implications in aspects of *grn1 *and *grn2 *function. Classically, antisense transcripts often function as inhibitors of the expression of their associated gene, through repression of transcription or promotion of mRNA degradation. There is the possibility that ASgrn1-2 is involved in the formation of hybrid grn wherein this complementary transcript may provide a scaffold to sequester grn1 and grn2 mRNA transcripts within the same intracellular locale, preventing or alternatively facilitating the *trans*-splicing reaction.

Regarding the RT-PCR data, despite evidence of clear tissue-specific and temporal ASgrn1-2 expression in adult tissues and various developmental stages respectively, this transcript shows no clear reciprocal relationship to grn1 or grn2 (Figures 6 and 7). These expression patterns were confirmed at all stages examined using sense riboprobes for grn1, grn2, and verified for specificity by using the corresponding sense riboprobe to hybrid grn as negative control (Figures 10 and 11, and data not shown). At 5 dpf, ASgrn1-2 is expressed in the presumed hyoid ossification center, stomach and rostral intestine, as well as swim bladder (Figure 11i–n). Although grn1 and grn2 are found within most of these tissues (particularly grn1), specific expression of ASgrn1-2 in the hyoid region undergoing osteogenesis may suggest the required down-regulation of the expression of its counterpart genes during bone development. Therefore a reciprocal relationship may exist between these genes under strict spatio-temporal regulation. Although ASgrn1-2 may mediate a degradation independent mode of gene regulation, such as alternative splicing, it clearly does not perform as a universal negative regulator of *grn1 *and *grn2 *expression.

At least one type of transcription modulation may be associated with ASgrn1-2 based on whole mount *in situ *hybridization analysis. There is a clear reciprocal relationship between hybrid grn expression and absence of ASgrn1-2 transcription. At 120 hpf, hybrid grn is restricted to the distal intestine and despite the expression of its substrate transcripts within several other locales, no other grn1/grn2 rich setting is devoid of ASgrn1-2 (Figure 11). This pattern further suggests that ASgrn1-2 expression prevents formation of the hybrid grn RNA.

The existence of *ASgrn1-2 *suggested that an equivalent entity may exist for one or both co-orthologues, *grna *and *grnb*. Indeed, the sense probe for grna in whole mount *in situ *hybridization analysis produced a consistent and reproducible signal within the intestine and pronephric ducts at 5 dpf (Figure 9, panel A, b). Several unidirectionally cloned cDNAs (accession numbers (CD585878, CD585963, and CD596001) were found to correspond to the reverse complement sequence of grna within the 3'UTR. Northern blot analysis using sense grna demonstrated a putative 4 kb transcript and directional cDNA synthesis followed by RT-PCR indicated this putative antisense transcript extended within the known grna intronic sequence. However, repeated attempts to clone the full-length transcript by RACE were unsuccessful. Nevertheless, these observations provide evidence for the existence of a second naturally occurring grn complementary transcript, namely ASgrna.

### The granulin motif – phylogenetic and functional implications

**Figure 12:**
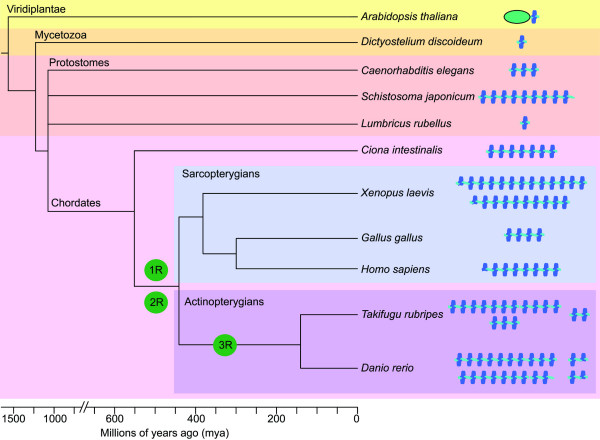
**Diagrammatic representation of the structures and evolutionary origins of the granulin in multicelluar organisms**. Evolutionary distances in millions of years derived from Hedges 2002 [77]. The estimated rounds of vertebrate genome duplication events are indicated (1R, 2R, 3R). The various progranulin structures were derived from various databases as outlined below. Land plant – (*Arabidopsis thaliana*) – Papain-like thiol protease bearing a carboxyl-terminal granulin domain (AAK71314); Slime mold – (*Dictyostelium discoideum*) – 1 copy progranulin from a single EST (AU267401) Trematode worm (*Schistosoma japonicum*) – 9 copy progranulin built from a combination of four ESTs (AY810079, BU790215, BU799560, BU771494); Nematode worm – (*Caenorhabditis elegans*) – 3 copy progranulin from single EST (NM_060580) – overall architecture confirmed by genome sequence (Z81595); Annelid worm (earthworm – *Lumbricus rubellus*) – 1 copy progranulin from a single EST (CO046860); Primitive chordate – (Sea squirt, *Ciona intestinalis*) – 7 copy progranulin predicted from draft genomic sequence (AABS01000126) and overall architecture confirmed by ESTs. Domains 2 to 7 nearly identical (BW368775, BW311239). Amphibian – (Frog, *Xenopus laevis*) co-orthologues progranulins A and B consisting of one-half domain followed by 12 full domains and one-half domain followed by nine full domains respectively [78]. These are structurally closely related and are a result of a recent tetraploidization event 30 mya [79]. Avian (chicken – *Gallus gallus*) – 4 copy progranulin built from three ESTs (BM440305, BU297352 and BX265765) – overall architecture confirmed by genome sequence (LOC426606); Human (*Homo sapiens*) – Progranulin composed of one half domain followed by 7 domains (UniProt entry: P28799); Teleost – *Takifugu rubripes *– 2, 3 and 11 copy progranulins predicted from draft genome sequences (M000077, S002118, S0001020) with overall structures confirmed by ESTs (CA846088, CA332411, AL842916, CA588603). Teleost – *Danio rerio*: from this study: co-orthologue progranulins A and B composed of 10 (NM_001001949) and 9 (AY289606) respectively; two smaller progranulins consisting of 1 and one half granulin repeats (AF273479, AF273480).

The granulin motif is so distinctive that it is a relatively simple matter to search the protein and nucleotide databases to obtain an impression of its phylogenetic origins. Figure 12 shows a diagrammatic representation of the nature of proteins bearing the granulin motif that can be confidently predicted through searching the cDNA, EST and/or genome sequence databases using BLAST [24]. The numbers of full granulin modules present in each putative protein is shown in cartoon form together with an impression of the phylogenetic origins of the species involved. The source of the sequence data used to predict these structures is listed as accession numbers in the legend to Figure 12. The distribution of some of the proteins bearing the granulin motif within assorted species has been partially annotated and can be viewed at various web sites [63,64]. With some interesting exceptions (e.g. *Drosophila melanogaster*), the granulin motif can be found throughout eukaryotes, both in plants and multicellular animals. In contrast there is no representation within fungi or unicellular organisms. The presence of a protein bearing the granulin motif within the slime mold (*Dictyostelium discoideum*) is particularly revealing since this organism is thought to be a modern representative of an amoeboid organism that was a transition species between unicellular and multicellular eukaryotes [65]. The appearance of this organism is thought to predate the divergence of plants and animals about 1.5 billion years ago. This suggests that a primordial gene bearing a single granulin motif evolved once during this transition period. The plant motif is found in just one context as a carboxyl-terminal domain of a cysteine protease that is found in many members of the viridiplantae including *Arabidopsis thaliana *[66] (Figure 12). Together this suggests that the founding granulin gene was represented by a single copy and composed of a single granulin motif.

In contrast to the conserved protease/granulin gene architecture found in plants, members of the animal kingdom have expanded their granulin repertoire, not via genomic or segmental duplication, but rather through tandem multiplication of the granulin ORF. For instance the *Xenopus leavis *and the early chordate *Ciona intestinalis *granulin genes encode of five and six tandemly repeated near identical granulin motifs, respectively. The variance in the number of granulin intragenic regions demonstrates a degree of plasticity during *grn *gene expansion. Intragenic multiplication is unlikely to have occurred as a single ancestral event; rather *grn *gene expansion has taken place independently in various species to varying extents. This mechanism of conserved domain repetition is not unique to *grn *genes. Indeed, the same type of genetic expansion is likely responsible for the repetition of immunoglobulin, EGF and lectin domains in numerous proteins.

The presence of single *grn *genes in protostomes is not surprising since the origins of these species predate the estimated genomic duplications within the vertebrate radiation (1R and 2R, Figure 12); these large-scale events appear not to have given rise to *grn *gene expansion. Specifically, all members of the sarcopterygian lineage (derived from lobe-finned fish) harbour a single granulin gene of varying motif number, indicating that intragenic multiplication remains the preferred and tolerated means of granulin expansion for most vertebrates, including mammals. The same intolerance to gene duplication has not encompassed the actinopterygians (ray-finned fish), including *Danio rerio *and *Takifugu rubripes*. These species have undoubtedly expanded their granulin gene repertoire through tandem intragenic expansion as well as genome duplication (3R, Figure 12), to yield the co-orthologues *grna *and *grnb*. Interestingly, the existence of *grn1 *and *grn2 *does not necessarily conform to these methods of gene expansion, suggesting that a specific and unidentified means of gene expansion, possibly involving *ASgrn1-2*, may exist.

## Conclusion

Although the existence of two co-orthologues of mammalian progranulin in zebrafish is likely a result of genome-wide duplication, similar genetic events have occurred within chordates prior to divergence of the ray-finned (teleost) and lobe-finned (mammalia) radiations. Indeed two rounds of genome-wide duplication are believed to have occurred [67]. More precisely, the first genome duplication probably occurred in a common ancestor of all agnathans and gnathostomes after its divergence from cephalochordates, ~594 mya (million years ago). The second round is presumed to have occurred ~488 mya, within the lineage leading to jawed vertebrates after the jawless line diverged, presumably before the split between cartilaginous and bony fish. Despite this, all mammals studied thus far have retained only a single copy of the progranulin gene, whereas two rounds of genome duplication would theoretically create four progranulin genes. It is therefore interesting to consider the biological rationale behind retention of *grna *and *grnb *following the teleost genome duplication, an event not permitted within other vertebrates, in conjunction with the appearance of two extra paralogues, *grn1 *and *grn2*. Regulation by gene dosage through complementary transcription may have allowed for the retention of the smaller paralogues, while putative antisense transcription to grna may be necessary for precisely regulating the spatio-temporal activity of this growth factor.

Overall, the expression patterns of zebrafish progranulins faithfully replicate those observed for the mouse counterpart in a similar context [42,46]. Importantly, this indicates that the use of zebrafish will enable modeling of the contributions of progranulin activity to vertebrate development through investigating both *grna *and *grnb*. These studies will be uncomplicated by the presence of *grn1 *and *grn2*, whose expression patterns largely do not overlap with the co-orthologues. Overall, the expression patterns for the *grns *indicate that these growth factors may subserve multiple functions *in vivo *that are consistent with the known role of their mammalian counterpart in cell growth, motility and survival.

## Materials and methods

### Tissue extraction and granulin peptide purification

Carp (*Cyprinus carpio*) were purchased live at a local fish market (Waldman Plus, Montreal, QUE). Peptides from fish spleens were extracted using C_18 _Sep-Pak cartridges (Waters Canada Ltd. Mississauga, ONT) and separated using reversed-phase high-performance liquid chromatography (RP-HPLC) on a C_18 _Bondapak column (Waters) as previously described [19,68]. Column fractions were screened for cysteine content by amino acid analysis and granulin-1/2 immunoreactivity by radioimmunoassay [19]. Fractions positive for both criteria were further purified by RP-HPLC using solvents containing 0.13% (v/v) heptafluorobutyric acid as counter-ion, and subsequently purified to homogeneity using the original solvent system containing 0.1% (v/v) trifluoroacetic acid. Molecular weight of purified peptides was determined using a Voyager matrix-assisted laser desorption ionization time-of-flight (MALDI-TOF) mass spectrometer (Perceptive Biosystems, Framingham, MA) located at the Sheldon Biotechnology Centre of McGill University.

### Microsequencing of carp granulin-A

The putative carp granulin-A was alkylated according to a previously published protocol [19]. Approximately 20 μg of the alkylated carp granulin-A was digested with sequencing grade chymotrypsin (Roche Diagnostics Canada, Laval, QUE) according to the manufacturer's instructions, and the resulting fragments separated by RP-HPLC on a C_18 _Bondapak column. Amino-terminal sequence analysis of carp granulin-A and its chymotryptic fragments was undertaken using a Procise sequencer (Applied Biosystems, Foster City, CA) located at the Sheldon Biotechnology Centre of McGill University.

### Fish husbandry

Wild type zebrafish were purchased from Scientific Hatcheries (Huntington Beach, CA) and maintained on a 14 h/10 h light/dark cycle at 28.5°C in a laboratory aquarium (Allantown Aquaneering, Allantown, NJ). Fish were fed twice daily, and bred as described elsewhere [69]. Embryos for developmental studies were collected from tanks and staged according to conventional criteria [70] and by hours post-fertilization (hpf).

### Library screening and cloning of zebrafish progranulins

The zebrafish grn1 cDNA was cloned using a PCR strategy (Additional File 1). The carp granulin-1 amino acid sequence was used to design degenerate forward DF1 (5'-GTI ATY CAY TGY GAY GC-3') and reverse DR1 (5'-CAR CAR TGR ATI CCR TC-3') and DR2 (5'-TCR CAR TGR TAI CCR TG-3') primers for use in the polymerase chain reaction (IUPAC codes are used to refer to the bases in primer sequences). The template for the initial amplification reaction was a 5'-STRETCH plus cDNA library cloned in lambda gt10 vector (Clontech BD Biosciences, Mississauga, ONT). cDNA for this library was prepared from 1-month-old zebrafish using a combination of oligo-dT and random priming. 0.25 μl of library (approximately 10^8 ^pfu/ml) was used in a final reaction volume of 100 μl for each new amplification attempt. The annealing temperature was determined empirically in order to maximize yield of product. PCR amplifications were performed with Taq DNA polymerase, unless specified otherwise, using a Hybaid thermal cycler from Bio/Can Scientific Inc. (Etobicoke, ONT). Amplified products were isolated by agarose gel electrophoresis, purified with the QIAquick Gel Extraction Kit (Qiagen Inc. Mississauga, ONT) and sequenced after cloning into TOPO pCR2.1 (Invitrogen, Carlsbad, CA). An initial reaction using the DF1 and DR1 primer pair yielded several products. 5 μl of this reaction was subjected to re-amplification using DF1 primer in combination with the nested (anchored) DR2 primer, which revealed a product of 126-bp encoding a partial sequence for granulin-1 (Additional File 1, step 1). New grn1 primers F126 (5'-ACTGTGTGTCCAGACGG-3') and R215 (5'-CCATCCCTGCAACACTG-3') were then designed based on this sequence and were used, respectively, in combination with flanking gt10 primers in order to obtain the 5'- and 3'-untranslated region (UTR) cDNA sequences (Additional File 1, steps 2 and 3). Finally, the entire ORF was amplified with Pwo DNA polymerase (Roche Diagnostics), using forward F1 (5'-ATGTTCCCAGTGTTGATG-3') and reverse R (STOP) (5'-GCTTACAACTCCAACCCG-3') primers (Additional File 1, Step 4). This PCR was performed in a final volume of 100 μl, containing 0.25 μl of library, 50 mM KCl, 10 mM Tris-HCl, (pH 8.8), 1.5 mM MgCl_2_, 0.1% Triton X-100, 0.2 mM concentration of each dNTP, 0.5 unit of Pwo DNA polymerase, and 100 pmol of each primer. An initial denaturation step was carried out at 94°C for 3 min. Annealing temperatures of 54°C, 56°C and 58°C were used sequentially for 10 cycles each. Typical denaturation, annealing, and amplification reactions were carried out at 94°C for 30 sec, 54°C for 1 min, and 68°C for 1 min, respectively. A final extension step of 10 min at 72°C was carried out after adding 0.25 unit Taq DNA polymerase. An amplification product specific for grn1 was sequenced on both strands. The 5'-UTR, and a portion of the 3'-UTR for grn1, were amplified using grn1-specific primers in conjunction with a lambda gt10 primer. Distinct cDNAs encoding progranulin-2 and a chimeric progranulin were uncovered through this approach. Each transcript was confirmed through sequencing of independent amplification reactions using template cDNA derived from either adult organs or embryos of mixed stages. Following a strategy similar to that used for the isolation of zebrafish grn1 cDNA, primers based on the purified carp granulin-A peptide sequence were designed to clone partial cDNAs for zebrafish grna and grnb, respectively (not shown). BLAST searches using the cloned sequences retrieved two unique ESTs at NCBI sharing an exact match with grna and grnb, respectively (accession numbers AW174591 and AW184435). These respective ESTs were purchased from RZPD GmbH (Heidelberg, Germany; clone ID: UCDMp574E2318Q2 and UCDMp574I0223Q2) and sequenced on both strands to create a final assembly of the full-length cDNAs encoding zebrafish progranulin-A and progranulin-B. In addition to our cloning strategy, the rapid amplification of cDNA ends (RACE) was performed with the GeneRacer kit (Invitrogen, Burlington, ONT) using total RNA isolated from adult zebrafish intestine. For grn1 and grn2 transcripts, a reverse primer that corresponded to nucleotides 195–215 based on the cloned ORF of both transcripts (5'-CCATCCCTGCAACACTGACCC-3'), was used to perform the 5' RACE, while a forward primer corresponding to nucleotides 1–22 of each transcript (5'-ATGTTCCCAGTGTTGATGTTAC-3') was used to perform the RACE in the 3' direction. Similarly a 5' UTR sequence for grna was obtained using the map reverse primer (see below). Repeated RACE attempts in both directions for ASgrna were unsuccessful.

### Cloning of the zebrafish grn1 gene

A zebrafish genomic library constructed in P1 artificial chromosome (PAC) [71] and represented on filters at high-density (RZPD GmbH) was screened for the presence of the *grn1 *gene using standard procedures. The cDNA bearing the *grn1 *ORF was labeled with [α-^32^P] dCTP by random priming using the Oligolabeling kit (Amersham Biosciences, Baie d'Urfe, QUE) for use as probe, and purified using a Sephadex G-50 column (Amersham Biosciences). Kodak X-OMAT AR film was used for autoradiography (Fisher Scientific Ltd, Whitby, ONT). Three positive clones (706K2254Q, BUSMP706K14116Q2, 706F20133Q2) were detected by autoradiography, and the first two were confirmed to carry at least part of the grn1 gene by PCR, using the F1 (5'-ATGTTCCCAGTGTTGATG-3') and R215 (5'-CCATCCCTGCAACACTG-3') primer pair which does not discriminate between grn1 and grn2, and sequencing. DNA from a positive clone (706K2254Q) was purified with the Plasmid Midi Kit (Qiagen). 1.5 μg of this DNA was subjected to restriction digest with EcoRI to generate fragments suitable for cloning into pBluescript II KS (Stratagene, La Jolla, CA), and was followed by transformation in TOP 10F' electrocompetent cells (Invitrogen). Screening of colonies transferred onto nitrocellulose membranes (Xymotech, Montreal, QUE), employing the same probe used for the original library screening, was performed in the following prehybridization and hybridization conditions: 2 × SSC, 0.5% SDS, 0.05% Na Pyrophosphate at 65°C. Membranes were washed twice in 1 × SSC, 0.1% SDS, 0.05% Na Pyrophosphate at 60°C for 15 min, followed by two washes in 0.1% SSC, 0.1% SDS, 0.05% Na Pyrophosphate at 60°C for 10 min. Plasmid DNA from a positive clone was purified using the high pure plasmid isolation kit (Roche Diagnostics, Laval, QUE). An insert of ~9-kb was fully sequenced and revealed the presence of the promoter region and approximately half of the grn1 gene. The remaining gene sequence was found in a ~6-kb insert clone isolated by re-screening the colony lifts with ^32^P-labeled reverse R(STOP) oligonucleotide (5'-GCTTACAACTCCAACCCG-3') as probe. A PCR was performed using primers flanking this EcoRI site, and sequenced to confirm that the isolated 9 kb and 6 kb clones represent continuous sequences within the original PAC clone.

### Retrieval of antisense transcripts from NCBI

While in the process of analyzing cloned *grn1 *genomic sequences (data not shown) through BLAST searches for corresponding sequences at GenBank, an EST harbouring sequences corresponding to unspliced *grn1*, but in the reverse complement orientation, was noticed. This clone (accession number AW777232) was purchased (RZPD, clone ID: DKFZp717B091Q2) and further analyzed through sequencing. A putative transcript exhibiting perfect complementarity to the 3'UTR region of zebrafish grna (*ASgrna*) was deduced from sequencing four unidirectionally cloned ESTs deposited at GenBank (CD585878, CD585963, CD596001 and CD588938) that originated from an oligo-dT-primed cDNA synthesis from adult kidney marrow RNA (Song *et al*. 2004; kindly provided by Dr. Chen, Shanghai Institute of Biological Science). Unidirectional cDNA synthesis using total RNA derived 5 day-old larvae was synthesized using a sense primer relative to the 3'UTR exon (ASgrna 2) or to a known intron (ASgrna 3) of grna (Additional File 10 and 11). These primers were then used in conjunction with the following primer (ASgrna 1) in subsequent RT-PCR to confirm antisense transcription to grna.

### Chromosomal assignment and syntenic analysis

Zebrafish *grns *were mapped using the LN54 Radiation Hybrid Panel as previously described [29]. Primers for each gene are noted in Additional File 9. Each PCR reaction was carried in a final volume of 20 μl containing 100 ng "hybrid DNA", 500 mM KCl, 100 mM Tris-HCl (pH 8.3), 15 mM MgCl_2_, 0.2 mM each dNTP, 1 unit *Taq *DNA polymerase, and 5 pmol of each oligo. Denaturation, annealing and amplification were performed at 94°C for 30 sec, 55°C (*grn1 *and *grn2*) or 60°C (*grna *and *grnb*) for 30 sec, and 72°C for 30 sec, respectively, followed by an extension step of 7 min at 72°C. To determine syntenic relationships between zebrafish and human genomes, mapped zebrafish genes flanking a given zebrafish *grn *gene were identified using the consolidated zebrafish maps available from ZFIN [72] and data from LocusLink [73].

### Gene expression profiling by RT-PCR

Total RNA from various adult tissues and developmental stages was isolated using Trizol LS reagent (Gibco BRL, Burlington, ONT), treated with DNaseI and used in first strand synthesis using the Revert Aid H-synthesis kit (MBI Fermentas Inc. Burlington, ONT). PCR conditions used for each family member consisted of an initial denaturation at 94°C for 2 min, followed by 30–40 cycles at 94°C for 45 sec, gene-specific annealing temperature (Additional File 10) for 1 min and extension at 72°C for 1 min, with a final single cycle extension at 72°C for 7 min. To discriminate between *grn1*, *grn2 *and *hybrid grn*, an (NH_4_)_2_SO_4 _buffer with 1 mM MgCl_2 _(MBI Fermentas) was used for RT-PCR in conjunction with cloned template controls (pBluescript, Clonetech) for all three forms using the same reaction parameters. Two independent reverse primers for *grn1 *and *grn2 *yielded products of expected size and *hybrid grn *was produced using *grn1 *forward with either of the *grn2 *reverse primers. All PCR products were resolved on 2% agarose gels, ethidium bromide stained and visualized on Polaroid 667 Film. The authenticity of all PCR products was confirmed by sequencing after cloning into TOPO/pCR2.1.

### Northern blot analysis

Full-length mRNA transcript size was assessed for each progranulin family member by Northern analysis of poly-A enriched mRNA (Micro Poly (A) Purist Small Scale Purification Kit; Ambion) derived from whole adult or 5 dpf animals [74]. Hybridization (Ultrahyb; Ambion, Austin, TX) was carried out using non-radioactive biotin-labeled cRNA probes (Psoralen-Biotin Non-iosotopic labeling Kit; Ambion) and detected with Brightstar BioDetect Nonisotopic Detection Kit (Ambion) according to manufacturers instructions. Band size was determined using pre-labeled biotin markers (Ambion).

### Whole-mount mRNA *in situ *hybridization

*In situ *hybridization for progranulin family gene expression was carried out essentially as previously described [75]. Briefly, digoxigenin-labeled RNA probes for each full-length cDNA, with the exception of ASgrn-1/2 which corresponded to exons 1–3 only, were hybridized at 70°C using various developmental stages from cleavage to larval. In some cases, polyvinyl alcohol was added to the staining solution in order to minimize the occurrence of background, especially when the reaction was required to proceed for several days [76]. Stained whole-mount and sectioned embryos were mounted in glycerol and visualized under a Leica MZFLIII stereomicroscope (Richmond Hill, ONT). Pictures were taken with a Leica DC350F camera and processed with Adobe Photoshop 7.0 software.

## Sequence Accession Numbers

GenBank accession numbers of all zebrafish proranulin genes and antisense transcripts described in this paper are as fellows: *grn1*, AF273479; *grn2*, AF273480; *hybrid grn*, AF273481; *ASgrn1-2*, AY289607; *grna*, AF375477, *ASgrna*, AY826190; *grnb*, AY289606 .

## List of abbreviations

AER, apical ectodermal ridge; ASgrn, antisense granulin; CNS, central nervous system; DDC, duplication-degeneration-complementation; EVL, enveloping monolayer of cells; grn, granulin; hpf, hours post-fertilization; HSC, hematopoietic stem cell; ICM, intermediate cell mass; LG, linkage group; LPM, lateral plate mesoderm; mya, million years ago; SSLP, single sequence length polymorphism; YSL, yolk syncitial layer.

## Authors' contributions

Benoît Cadieux established and justified the zebrafish animal model, carried out all the molecular genetic studies and prepared the initial draft of the manuscript. Babykumari P. Chitramuthu carried out the whole-mount *in situ *hybridization studies and Northern blot analysis, participated in the design of the study and helped draft the manuscript, David Baranowski participated in the design of the study and helped draft the manuscript. Hugh P. J. Bennett conceived of the study, co-ordinated its design and helped draft and finalize the manuscript. All co-authors read and approved the final manuscript.

## Supplementary Material

Additional File 1 **Cloning strategy for the cDNA encoding the precursor for zebrafish granulin-1**. *Panel A*: The full-length cDNA for progranulin-1 is represented at the top of the diagram. Black rectangles represent the ORF, and blank rectangles represent the respective 5' and 3' untranslated regions. The dashed lines represent lambda phage (vector) sequences. Numbers on the left represent the sequential order of PCRs undertaken (see Materials and Methods section). *Panel B*: Deduced amino acid sequence for the precursor encoding granulin-1, consisting of one and one-half repeats of the granulin consensus motif. Characteristic cysteines are underlined and in bold. A predicted leader sequence is shown in italics. The full granulin-1 peptide sequence (35–91) is separated from the amino-terminal half peptide (116–147) by an intervening sequence. Stop codon is represented by *. Numbers represent amino acid position. Click here for file

Additional File 2 **Genomic architecture of the zebrafish grn1 gene**. Exons (1–5) and deduced amino acid sequences are shown in uppercase letters and are boxed, while introns (A–D) and flanking sequences are shown in lowercase. 5' extension of exon-1 is based on a deposited EST sequence (accession number BG884011) and 5' RACE. A potential TATA box in the promoter region is in bold and italics. The translation initiation codon (ATG) and polyadenylation signal sequence (AATAAA) are in italics. The EcoRI restriction site (gaattc) used for cloning the genomic fragments for this gene is located in intron C, and is underlined and bold. An identical exonic architecture was found for grn2 (data not shown). Click here for file

Additional File 3 **Conserved exonic organization of zebrafish grn1 and grn2 genes relative to mammalian progranulin**. Like granulin repeat units found in mammalian progranulin the nucleotide sequences encoding zebrafish granulin-1 (and granulin-2, not shown here) is derived from the joining of two spliced exons with phase 0 boundaries. This characteristic splicing occurs at nucleotide positions corresponding to four amino acids after cysteine 6 within the amino-terminal region (exon 2), and two amino acids before cysteine 7 in the carboxyl-terminal end (exon 3). The relative sizes of exons (1–5) and introns (A–D) is indicated. Click here for file

Additional File 4 **Splice junctions of the zebrafish *grn1*, *grn2 *and the non-protein coding *ASgrn1-2 *genes**. The consensus sequence for splice donor and acceptor sites is shown on the top line (Breathnach and Chambon, 1981). The nucleotide sequences surrounding the sites for introns A–D for the respective *grn1 *and *grn2 *genes, as well as for introns A–C of the *ASgrn1-2 *gene, are shown. Exons are in uppercase, introns in lowercase. Phase of introns interrupting open reading frames are indicated. Click here for file

Additional File 5 **Cloning of a chimeric transcript that encodes a hybrid progranulin**. *Panel A*: Nucleotide sequence alignment of the ORFs for progranulin-1, chimeric progranulin, and progranulin-2. RACE confirmed that the cloned cDNAs possess identical 5' and 3' untranslated regions (not shown). After verifying the cDNA nucleotide sequences for grn1 and grn2 with corresponding exonic segments (boxed), it was found that for the chimeric (hybrid grn) transcript, all except one nucleotide substitution (T instead of C, exon 4), are conserved and non-randomly distributed among corresponding exonic sequences of either the grn1 (highlighted in blue, exon 2) or grn2 (highlighted in orange, exons 3–5) genes. The translation initiation (ATG) and termination (TAA) codons are in bold. Arrows indicate location of primers used for RT-PCR analyses (bold; see Additional File 10). *Panel B*: Sequence alignment of the deduced translated sequences for progranulin-1, progranulin-2, and hybrid progranulin. The candidate chimeric transcript consists of the amino-terminal portion of grn1 (exons 1 and 2) and of the carboxyl-terminal portion of progranulin-2 (exons 3 to 5). The position of introns (A–D) located in the respective grn1 and grn2 genes are indicated by arrowheads. The granulin-1 peptide and amino-terminal half-domain are underlined. Click here for file

Additional File 6 **Nucleotide sequence of a transcript antisense to zebrafish grn1 and grn2 genes**. The 1989 nucleotide ASgrn1-2 cDNA is encoded on four exons (boxed) and bears a polyadenylation signal (AATAAA) (bold and italics). Sequences corresponding to exons 2 and 3 of ASgrn1-2 are complementary to regions encompassing the second and third exons (uppercase and bold) and intronic sequences (lowercase) of the grn1 and grn2 genes, respectively. The nucleotide sequence of exon 4 corresponds to a mutated transposase gene of the *tzf *transposon sub-class of the Tc1/mariner superfamily of mobile elements, but in the reverse complement orientation. ASgrn1-2 is a non-protein coding RNA based on the absence of a predictable open reading frame. Click here for file

Additional File 7 **The genomic region encompassing exon 4 of the ASgrn1-2 gene corresponds to a defective transposon of the tzf family, in the reverse complement orientation**. A consensus 1621 nucleotide sequence for the *tzf *transposon, deduced by the majority rule from aligned sequences obtained from GenBank EST entries (U51226-U51230) and published data (Lam et al. 1996), is shown on top in uppercase, with the characteristic 200 nucleotides inverted repeats (underlined) and terminal TA dinucleotides (bold). The translation initiation (ATG) and termination (TGA) codons of the transposase gene are in bold and boxed. The reverse complement sequence of the region encompassing exon 4 of the ASgrn1-2 gene (boxed) is aligned underneath the transposon sequence, with mismatches represented in lowercase. Deletions (-) and insertions (+) relative to the intact transposon sequence that have occurred within exon 4 and flanking intronic sequences of the ASgrn1-2 gene, rendering this mobile element inactive, are indicated. Click here for file

Additional File 8 **Northern analysis of zebrafish grn sense and antisense transcripts**. Northern blot analyses using strand-specific cRNA probes were performed to confirm the size of cloned cDNA sequences and resolve the issue of antisense transcription to grna. *Panel A*: grna is expressed as a predominant transcript of 3.7 kb in size, consistent with cloned sequences (3649 bp), and can also be found as a larger transcript exceeding 6 kb. *Panel B*: Four grnb transcripts of approximately 1.5 kb, 1.8 kb, 2.9 kb and 5.2 kb in size, respectively, are detected. Note that the 2.9 kb band, showing strongest intensity, is in agreement with cloned sequences for this mRNA (2820 bp). *Panel C*: A riboprobe targeting ASgrna detects a faint transcript of approximately 4 kb in size, suggesting that the cloned sequences for this antisense transcript (914 bp) may represent a splice variant. *Panel D*: The grn1 transcript is approximately 0.8 kb in size, as expected from cloned sequences (see Additional Files 2 and 3), but also expressed at lower levels as a larger transcript of approximately 1.7 kb. *Panel E*: Unlike grn1, grn2 is not expressed as a transcript other than 0.8 kb in size. *Panel F*: A band of low intensity can be detected at 0.8 kb for the hybrid grn RNA, suggesting that no cross-hybridization occurs with grn1 and grn2 mRNAs using this riboprobe. The hybrid grn riboprobe also detects a band of approximately 1.7 kb similar in intensity to that seen for grn1. *Panel G*: The overall abundance of the ASgrn1-2 transcript is too low for detection. A–C: 15 μg poly-A enriched RNA; D–G: 15 μg total RNA, each derived from a whole adult. Click here for file

Additional File 9 **Primers used in the linkage group assignment of the zebrafish progranulin genes**. Before applying to the LN 54 mapping panel, conditions for each primer combination were optimized by PCR with the use of zebrafish genomic DNA derived from the AB wild type strain. The authenticity and specificity of each amplicon was verified by sequencing after cloning into the pCRII plasmid. For the assignment to zebrafish linkage groups, each PCR amplification experiment was performed at least twice. Click here for file

Additional File 10 **Sequences of primers and predicted sizes of PCR amplicons**. All primer pairs are located on consecutive exons of their corresponding gene. Click here for file

Additional File 11 **Partial characterization of a putative antisense transcript to zebrafish grna**. *Panel A*: Schematic representation of the location of ASgrna, a unidirectionally cloned transcript sharing complementarity with part of the last exon of the zebrafish grna gene. The relative position of primers used for cDNA synthesis (primers 2 and 3, respectively) and subsequent RT-PCR are indicated (see Materials and Methods). *Panel B*: RT-PCR analysis of cDNA synthesized using either primer 2 or primer 3, respectively, with (+) or without (-) reverse-transcriptase. Primers depicted in *A *are used in the following combination for the RT-PCR: pair 1/2 (342 bp product) and pair 1/3 (478 bp product). The presence of an amplicon for both primer pairs using each +RT cDNA template, but not using template derived from -RT reactions, indicates that ASgrna extends in the 3'end direction and overlaps with intronic sequences of grna. Positive controls for PCR conditions using genomic DNA as template, as well as a no template control are indicated, respectively. The authenticity of the amplicons was determined through sequencing. Each step of the experimental procedure was performed twice. Click here for file
