# Supplementary material for: The zebrafish progranulin gene family and antisense transcripts
Source: BMC Genomics. 2005 Nov 8;6:156. doi: 10.1186/1471-2164-6-156 (PMC1310530; doi:10.1186/1471-2164-6-156)
Supplement: Additional File 1 — Cloning strategy for the cDNA encoding the precursor for zebrafish granulin-1. Panel A: The full-length cDNA for progranulin-1 is represented at the top of the diagram. Black rectangles represent the ORF, and blank rectangles represent the respective 5' and 3' untranslated regions. The dashed lines represent lambda phage (vector) sequences. Numbers on the left represent the sequential order of PCRs undertaken (see Materials and Methods section). Panel B: Deduced amino acid sequence for the precursor encoding granulin-1, consisting of one and one-half repeats of the granulin consensus motif. Characteristic cysteines are underlined and in bold. A predicted leader sequence is shown in italics. The full granulin-1 peptide sequence (35–91) is separated from the amino-terminal half peptide (116–147) by an intervening sequence. Stop codon is represented by *. Numbers represent amino acid position. [file 1471-2164-6-156-S1.pdf]

A

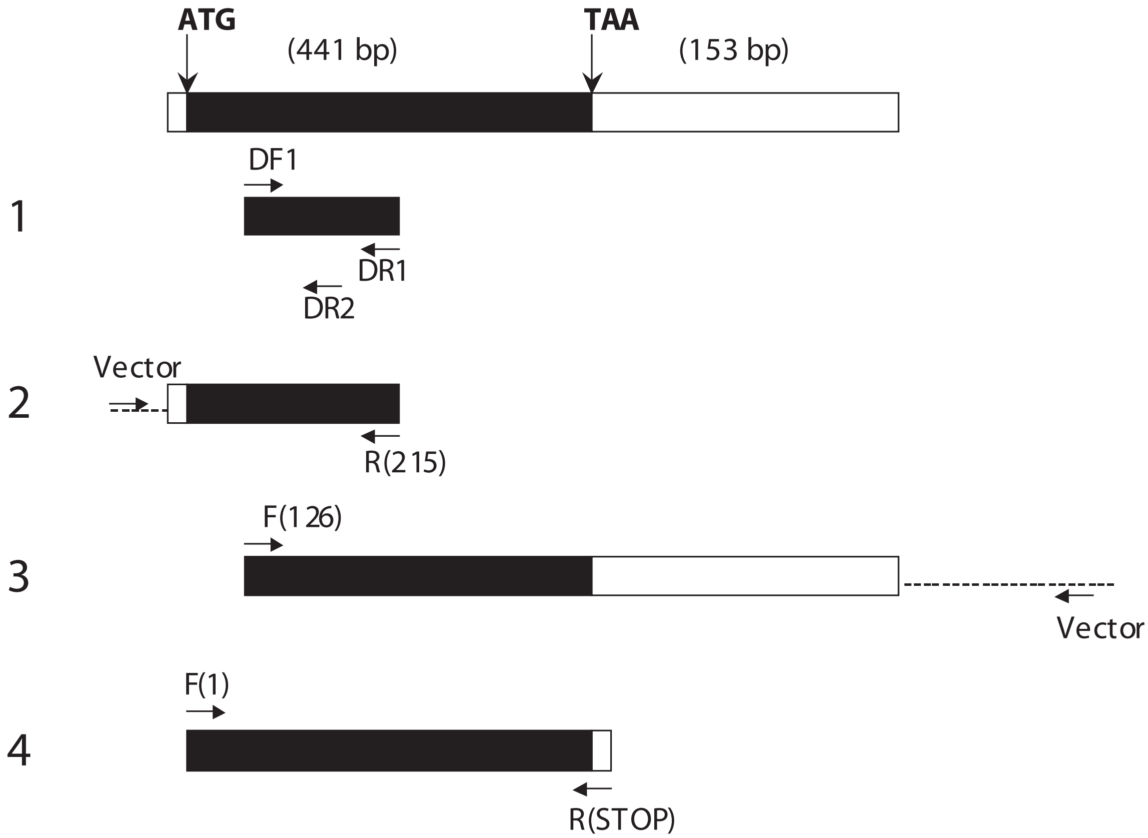

B

1 *MFPVLMLLMAALVAA*DEPLLDLSIPVETVDTSAS **34**
35 VIH**C**DAQTV**C**PDGTT**CC**LSPYGIWS**CC**PYSMGQ**CC**RDGIH**CC**QHGYR**C**DSTSTR**C**LR **91**
92 GWLTLPS SFQKATRTFQKDQTHAE **115**
116 TVQ**C**EGNFY**C**PAEKF**CC**KTRTGQWG**CC**SGLEL* **147**
