# Supplementary figures and images for: The zebrafish progranulin gene family and antisense transcripts
Source: BMC Genomics. 2005 Nov 8;6:156. doi: 10.1186/1471-2164-6-156 (PMC1310530; doi:10.1186/1471-2164-6-156)

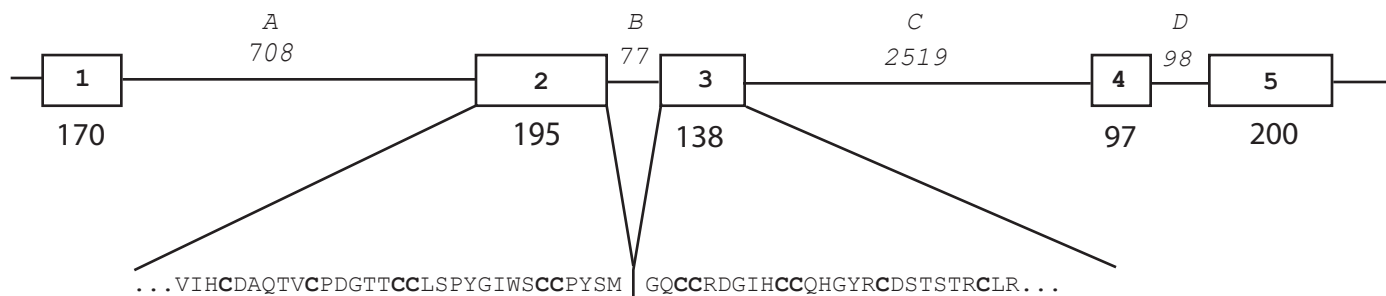

Supplement: Additional File 3 — Conserved exonic organization of zebrafish grn1 and grn2 genes relative to mammalian progranulin. Like granulin repeat units found in mammalian progranulin the nucleotide sequences encoding zebrafish granulin-1 (and granulin-2, not shown here) is derived from the joining of two spliced exons with phase 0 boundaries. This characteristic splicing occurs at nucleotide positions corresponding to four amino acids after cysteine 6 within the amino-terminal region (exon 2), and two amino acids before cysteine 7 in the carboxyl-terminal end (exon 3). The relative sizes of exons (1–5) and introns (A–D) is indicated. [file 1471-2164-6-156-S3.pdf]

A

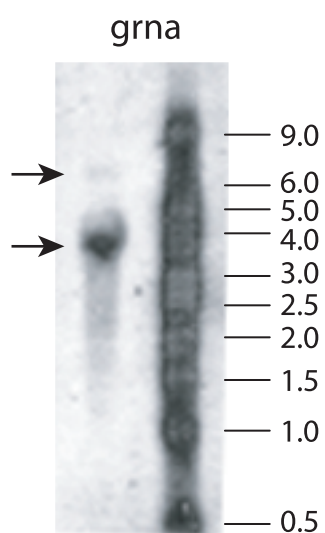

B

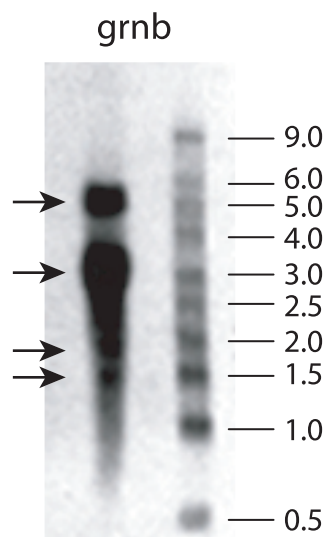

C

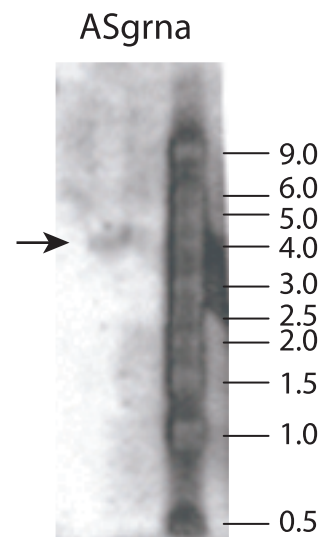

D

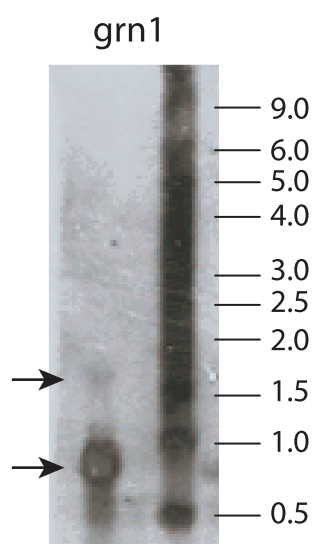

E

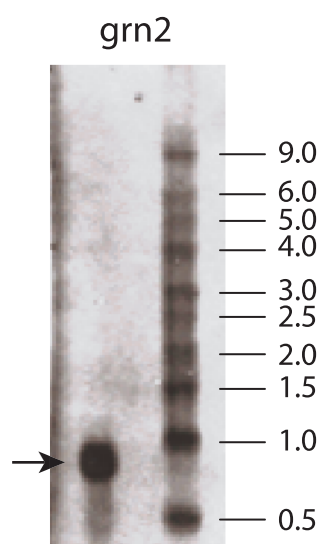

F

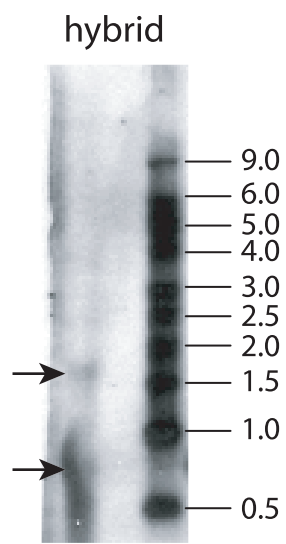

G

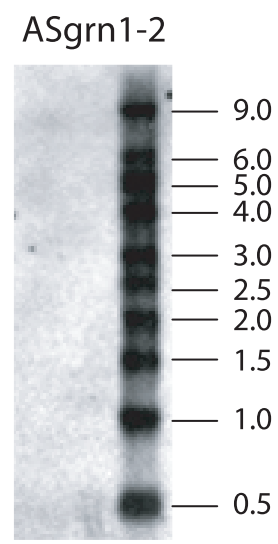

Supplement: Additional File 8 — Northern analysis of zebrafish grn sense and antisense transcripts. Northern blot analyses using strand-specific cRNA probes were performed to confirm the size of cloned cDNA sequences and resolve the issue of antisense transcription to grna. Panel A: grna is expressed as a predominant transcript of 3.7 kb in size, consistent with cloned sequences (3649 bp), and can also be found as a larger transcript exceeding 6 kb. Panel B: Four grnb transcripts of approximately 1.5 kb, 1.8 kb, 2.9 kb and 5.2 kb in size, respectively, are detected. Note that the 2.9 kb band, showing strongest intensity, is in agreement with cloned sequences for this mRNA (2820 bp). Panel C: A riboprobe targeting ASgrna detects a faint transcript of approximately 4 kb in size, suggesting that the cloned sequences for this antisense transcript (914 bp) may represent a splice variant. Panel D: The grn1 transcript is approximately 0.8 kb in size, as expected from cloned sequences (see Additional Files 2 and 3), but also expressed at lower levels as a larger transcript of approximately 1.7 kb. Panel E: Unlike grn1, grn2 is not expressed as a transcript other than 0.8 kb in size. Panel F: A band of low intensity can be detected at 0.8 kb for the hybrid grn RNA, suggesting that no cross-hybridization occurs with grn1 and grn2 mRNAs using this riboprobe. The hybrid grn riboprobe also detects a band of approximately 1.7 kb similar in intensity to that seen for grn1. Panel G: The overall abundance of the ASgrn1-2 transcript is too low for detection. A–C: 15 μg poly-A enriched RNA; D–G: 15 μg total RNA, each derived from a whole adult. [file 1471-2164-6-156-S8.pdf]

**A**

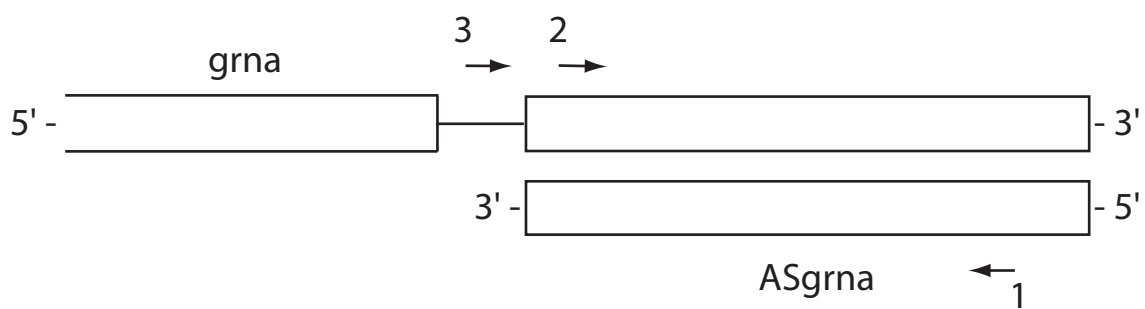

**B**

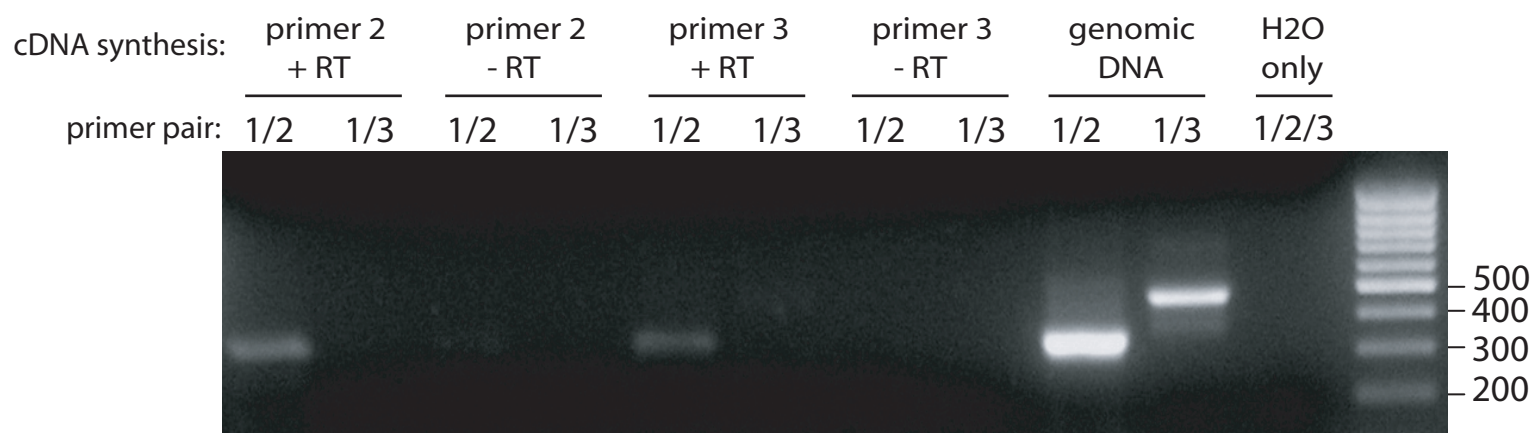

Supplement: Additional File 11 — Partial characterization of a putative antisense transcript to zebrafish grna. Panel A: Schematic representation of the location of ASgrna, a unidirectionally cloned transcript sharing complementarity with part of the last exon of the zebrafish grna gene. The relative position of primers used for cDNA synthesis (primers 2 and 3, respectively) and subsequent RT-PCR are indicated (see Materials and Methods). Panel B: RT-PCR analysis of cDNA synthesized using either primer 2 or primer 3, respectively, with (+) or without (-) reverse-transcriptase. Primers depicted in A are used in the following combination for the RT-PCR: pair 1/2 (342 bp product) and pair 1/3 (478 bp product). The presence of an amplicon for both primer pairs using each +RT cDNA template, but not using template derived from -RT reactions, indicates that ASgrna extends in the 3'end direction and overlaps with intronic sequences of grna. Positive controls for PCR conditions using genomic DNA as template, as well as a no template control are indicated, respectively. The authenticity of the amplicons was determined through sequencing. Each step of the experimental procedure was performed twice. [file 1471-2164-6-156-S11.pdf]
