# Supplementary material for: The zebrafish progranulin gene family and antisense transcripts
Source: BMC Genomics. 2005 Nov 8;6:156. doi: 10.1186/1471-2164-6-156 (PMC1310530; doi:10.1186/1471-2164-6-156)
Supplement: Additional File 4 — Splice junctions of the zebrafish grn1, grn2 and the non-protein coding ASgrn1-2 genes. The consensus sequence for splice donor and acceptor sites is shown on the top line (Breathnach and Chambon, 1981). The nucleotide sequences surrounding the sites for introns A–D for the respective grn1 and grn2 genes, as well as for introns A–C of the ASgrn1-2 gene, are shown. Exons are in uppercase, introns in lowercase. Phase of introns interrupting open reading frames are indicated. [file 1471-2164-6-156-S4.pdf]

consensus splice site sequences

5' donor (C/A)**AG / gt** (a,g)agt…..**INTRON**…… .(c,t)**ag / G** 3' acceptor

	splice phase
grn1	
(A)AG / gt (a) aat……. intron A………(c)ag / A	0
(A)TG / gt (a) aca……. intron B………(c)ag / G	0
(C)AG / gt (a) tgg……. intron C………(t)ag / A	0
(G)CG / gt (a) aaa……. intron D………(t)ag / G	1
grn2	
(A)AG / gt (a) aat……. intron A………(c)ag / A	0
(A)TG / gt (a) aga……. intron B ………(c)ag / G	0
(C)AG / gt (a) tgg…….intron C………(t)ag / A	0
(G)CG / gt (a) aaa……. intron D………(t)ag / A	1
ASgrn1-2	
(G)TG / gt (a) aga…. …intron A………(c)ag / T	N/A
(T)AG / gt (a) tgt…. . . . .intron B………(c)ag / T	
(T)AG / gt (a) tgt…. …. intron C………(c)ag / T	
