# Supplementary material for: The zebrafish progranulin gene family and antisense transcripts
Source: BMC Genomics. 2005 Nov 8;6:156. doi: 10.1186/1471-2164-6-156 (PMC1310530; doi:10.1186/1471-2164-6-156)
Supplement: Additional File 5 — Cloning of a chimeric transcript that encodes a hybrid progranulin. Panel A: Nucleotide sequence alignment of the ORFs for progranulin-1, chimeric progranulin, and progranulin-2. RACE confirmed that the cloned cDNAs possess identical 5' and 3' untranslated regions (not shown). After verifying the cDNA nucleotide sequences for grn1 and grn2 with corresponding exonic segments (boxed), it was found that for the chimeric (hybrid grn) transcript, all except one nucleotide substitution (T instead of C, exon 4), are conserved and non-randomly distributed among corresponding exonic sequences of either the grn1 (highlighted in blue, exon 2) or grn2 (highlighted in orange, exons 3–5) genes. The translation initiation (ATG) and termination (TAA) codons are in bold. Arrows indicate location of primers used for RT-PCR analyses (bold; see Additional File 10). Panel B: Sequence alignment of the deduced translated sequences for progranulin-1, progranulin-2, and hybrid progranulin. The candidate chimeric transcript consists of the amino-terminal portion of grn1 (exons 1 and 2) and of the carboxyl-terminal portion of progranulin-2 (exons 3 to 5). The position of introns (A–D) located in the respective grn1 and grn2 genes are indicated by arrowheads. The granulin-1 peptide and amino-terminal half-domain are underlined. [file 1471-2164-6-156-S5.pdf]

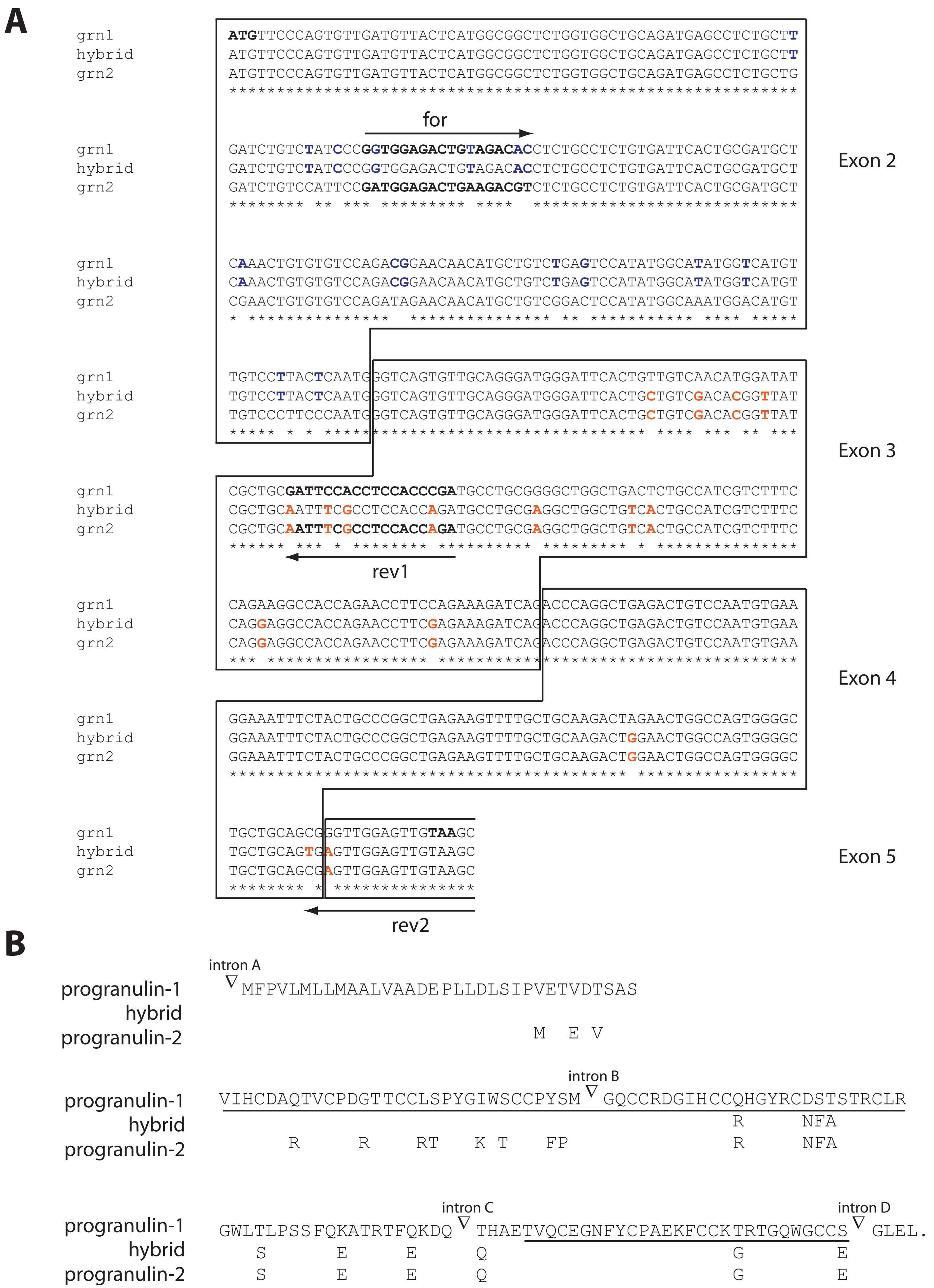
A
grn1 ATGTTCCCAGTGTTGATGTTACTCATGGCGGCTCTGGTGGCTGCAGATGAGCCTCTGCTT
hybrid ATGTTCCCAGTGTTGATGTTACTCATGGCGGCTCTGGTGGCTGCAGATGAGCCTCTGCTT
grn2 ATGTTCCCAGTGTTGATGTTACTCATGGCGGCTCTGGTGGCTGCAGATGAGCCTCTGCTG
for
grn1 GATCTGTCTATCCCGGTGGAGACTGTAGACACCTCTGCCTCTGTGATTCACTGCGATGCT
hybrid GATCTGTCTATCCCGGTGGAGACTGTAGACACCTCTGCCTCTGTGATTCACTGCGATGCT
grn2 GATCTGTCCATTCCGATGGAGACTGAAGACGTCTCTGCCTCTGTGATTCACTGCGATGCT
grn1 CAAACTGTGTGTCCAGACGGAACAACATGCTGTCTGAGTCCATATGGCATATGGTCATGT
hybrid CAAACTGTGTGTCCAGACGGAACAACATGCTGTCTGAGTCCATATGGCATATGGTCATGT
grn2 CGAACTGTGTGTCCAGATAGAACAACATGCTGTCGGACTCCATATGGCAAATGGACATGT
grn1 TGTCCTTACTCAATGGGTCAGTGTTGCAGGGATGGGATTCACTGTTGTCAACATGGATAT
hybrid TGTCCTTACTCAATGGGTCAGTGTTGCAGGGATGGGATTCACTGCTGTCGACACGGTTAT
grn2 TGTCCCTTCCCAATGGGTCAGTGTTGCAGGGATGGGATTCACTGCTGTCGACACGGTTAT
grn1 CGCTGCGATTCCACCTCCACCCGATGCCTGCGGGGCTGGCTGACTCTGCCATCGTCTTTC
hybrid CGCTGCAATTTCGCCTCCACCAGATGCCTGCGAGGCTGGCTGTCACTGCCATCGTCTTTC
grn2 CGCTGCAATTTCGCCTCCACCAGATGCCTGCGAGGCTGGCTGTCACTGCCATCGTCTTTC
rev1
grn1 CAGAAGGCCACCAGAACCTTCCAGAAAGATCAGACCCAGGCTGAGACTGTCCAATGTGAA
hybrid CAGGAGGCCACCAGAACCTTCGAGAAAGATCAGACCCAGGCTGAGACTGTCCAATGTGAA
grn2 CAGGAGGCCACCAGAACCTTCGAGAAAGATCAGACCCAGGCTGAGACTGTCCAATGTGAA
grn1 GGAAATTTCTACTGCCCGGCTGAGAAGTTTTGCTGCAAGACTAGAACTGGCCAGTGGGGC
hybrid GGAAATTTCTACTGCCCGGCTGAGAAGTTTTGCTGCAAGACTGGAACTGGCCAGTGGGGC
grn2 GGAAATTTCTACTGCCCGGCTGAGAAGTTTTGCTGCAAGACTGGAACTGGCCAGTGGGGC
grn1 TGCTGCAGCGGGTTGGAGTTGTAAGC
hybrid TGCTGCAGTGAGTTGGAGTTGTAAGC
grn2 TGCTGCAGCGAGTTGGAGTTGTAAGC
rev2
Exon 2
Exon 3
Exon 4
Exon 5
B
intron A
progranulin-1 MFPVLMLLMAALVAADEPLLDLSIPVETVDTSAS
hybrid
progranulin-2 M E V
intron B
progranulin-1 VIHCDAQTVCPDGTTCCLSPYGIWSCCPYSM GQCCRDGIHCCQHGYRCDSTSTRCLR
hybrid R NFA
progranulin-2 R R RT K T FP R NFA
intron C
intron D
progranulin-1 GWLTLPSSFQKATRTFQKDQ THAETVQCEGNFYCPAEKFCCKTRTGQWGCCS GLEL.
hybrid S E E Q G E
progranulin-2 S E E Q G E
