# Supplementary material for: The zebrafish progranulin gene family and antisense transcripts
Source: BMC Genomics. 2005 Nov 8;6:156. doi: 10.1186/1471-2164-6-156 (PMC1310530; doi:10.1186/1471-2164-6-156)
Supplement: Additional File 6 — Nucleotide sequence of a transcript antisense to zebrafish grn1 and grn2 genes. The 1989 nucleotide ASgrn1-2 cDNA is encoded on four exons (boxed) and bears a polyadenylation signal (AATAAA) (bold and italics). Sequences corresponding to exons 2 and 3 of ASgrn1-2 are complementary to regions encompassing the second and third exons (uppercase and bold) and intronic sequences (lowercase) of the grn1 and grn2 genes, respectively. The nucleotide sequence of exon 4 corresponds to a mutated transposase gene of the tzf transposon sub-class of the Tc1/mariner superfamily of mobile elements, but in the reverse complement orientation. ASgrn1-2 is a non-protein coding RNA based on the absence of a predictable open reading frame. [file 1471-2164-6-156-S6.pdf]

Antisense transcript to grn1 and grn2 (5'- 3')

exon 1

ATCGATTTAACGCATTTTAACTTGTCGAGCGTTTCCCTTCAGGGACGCATTTTGCAAATT
TATGAAGGTAATTCTTCGTTTAAAGTCGTATACCAGCGGATACAGTATGTG

exon 2

tggtctgccatac**CTGATCTTTCTGGAAGGTTCTGGTGGCCTTCTGGAAGGACGATGG**
CAGAGTCAGCCAGCCCCGCAGGCATCGGGTGGAGGTGGAATCGCAGCGATATCCATGTTG
ACAACAGTGAATCCCATCCCTGCAACACTGACCctgaagcagagaagagagtgaacaaag
gttaaccctgtaactatgagtagaaacatattaactcgagattctgttac**CATTGAGTAA**
GGACAACATGACCATATGCCATATGGACTCAGACAGCATGTTGTTCCGTCTGGACACACA
GTTTGAGCATCGCAGTAAATCACAGAGGCAGAGGTGTCTACAGTCTCCACCGGGATAGAC
AGATCAAGCAGAGGCTCATCTGCAGCCACCAGAGCCGCCATGAGTAACATCAACACTGGG
AACATctgaaaatgtgaaataggagagtattag

Reverse complement of genomic region encompassing exons 2 and 3 of **grn1**

exon 3

tggtctgccatac**CTGATCTTTCTCG**
AAGGTTCTGGTGGCCTCCTGGAAGGACGATGGCAGTGACAGCCAGCCTCGCAGGCATCGG
GTGGAGGCGAAATTGCACGATAACCGTGTCGACAGCAGTGAATCCCATCCCTGCAACACT
GACC ctgaagcagaggagagagtgagcaaaggttaaccctgtaactatgagtagaagaat
ataaaaaacaagattctcttac**CATTGGGAATGGACAACATGTCCATTTGCCATATGGAG**
TCCGACAGCATGTTGTTCTATCTGGACACACAGTTCGAGCATCGCAGTGAATCACAGAGG
CAGAGACGTCTTCAGTCTCCATCGGAATGGACAGATCCAGCAGAGGCTCATCTGCAGCCA
CCAGAGCCGCCATGAGTAACATCAACACTGGGAACATctgaaaatgtgaaataggagaat
ATTAG

Reverse complement of genomic region encompassing exons 2 and 3 of **grn2**

exon 4

TATCTCCCAAGCTCTATCAGGTTGGATGGGCAGCGACGATGTACAACCATTT
TCAGATCTCTCCAGAGATGTTTAATAGGATTTAGGTGTGGCGGTGTGCTTTGGGTCATTG
TCCTGCTGTAAGATGCCCCAGTCTGAGGTCAAGAGCACTCTGAAGCAGGTTTTCATTCAG
GATGTCTCTGTACATTGCTGCATTCATCTTTCCCTCTATCCTGACTAGTCTTCTAGTTCC
TGCTGCTGAAAAACATCCCCACACCACCATGCTTCACTGTAGGGGTGGGATTAGCCTTAT
GATAAGCGGTGTCTGGTTTTCTATAAACGTAACACCTGGTGTTCACTCCAAGAGTTCCAT
TTTAGTCTCATTAGACCCGAATTTTGTTTCTTATGGTCTGAGAGTCCTTCAGATGGGTTA
GTCCTAAAATCCAGGCGGGCCTCTACACCTAACACGCCTGAATGCTGGAGCTCAGACAGA
TTGACCATTGGGTAATTGATCACCCCCCTGACTAAGGCCCTTCTCCCCCGATCACTCAGC
TTAGATGGCCAGCCAGCTCTAGGAAGAGTCCTGGTGGTTCCAAACATCTTCCACTTATCG
ATGATGGAGGCCACTGTGCTCACTGGAACTTTCAGAGCAGCAGAAATCTTTCTGTAACCT
TCCACAGTCTTGTGGCTCAAGACAATCCTGCCTCGGAGGTCTACAGACAATTCCTTTGTC
TTCATGCTTGGTTTGTGCTTTGACATGCACTGTCAACCCTGGGACCCTATATAGACAGGT
GTATGTCTTTTCAAATCATGTCCAATTAACTGAATTTACCACAGGTGAACTCCAATTAAG
CTGCTGAAACATCTCAAGAATGATCAGTGGAAACAGAATGTACCTGAGCTCCATTTAGAG
CTTCGCGCAAAGGCTGTGAATACTGATGAACATCTGATTTTTCAGGTTTTCTATTTAT***AA***
TAAATTTGCAACAATTTCAAAAAAAAAAAAAAAAA

Reverse complement of a tzf transposase gene
