# Supplementary material for: The zebrafish progranulin gene family and antisense transcripts
Source: BMC Genomics. 2005 Nov 8;6:156. doi: 10.1186/1471-2164-6-156 (PMC1310530; doi:10.1186/1471-2164-6-156)
Supplement: Additional File 7 — The genomic region encompassing exon 4 of the ASgrn1-2 gene corresponds to a defective transposon of the tzf family, in the reverse complement orientation. A consensus 1621 nucleotide sequence for the tzf transposon, deduced by the majority rule from aligned sequences obtained from GenBank EST entries (U51226-U51230) and published data (Lam et al. 1996), is shown on top in uppercase, with the characteristic 200 nucleotides inverted repeats (underlined) and terminal TA dinucleotides (bold). The translation initiation (ATG) and termination (TGA) codons of the transposase gene are in bold and boxed. The reverse complement sequence of the region encompassing exon 4 of the ASgrn1-2 gene (boxed) is aligned underneath the transposon sequence, with mismatches represented in lowercase. Deletions (-) and insertions (+) relative to the intact transposon sequence that have occurred within exon 4 and flanking intronic sequences of the ASgrn1-2 gene, rendering this mobile element inactive, are indicated. [file 1471-2164-6-156-S7.pdf]

ASgrn1-2 exon 4 (reverse complement)

5'

```
TACAGTGCATCCGGAAAGTATTCATAGCGCTTCACTTTTTCCACATTTTTTATGTTACAGCC
                        g                     g     g    c
TTATTCCAAAATGGATTAAATTAGTTTATTTCCTCAACATTCTACACACAATACCCCATAAT
                    -               ag        t    a at   g
GACAATGTGAAAAAAGATTTTTTGAAATTGTTGCAAATTTATTAAAAATAAAAAACCTGAAA
               c-                         t     g
AATCATATGTGCATCAGTATTCACAGCCTTTGCCGTGAAGCTCTAAATTGAGCTCAGGTACA
     g    t                     - c            g
TTCTGTTTTCTAAATTGAGCTCAGGTACATTCTGTTTCCACTGATCATTCTTGAGATGTTTC
               g
AGCAGCTTCATTGGAGTTCACCTGTGGTAAATTCAGTTAATTGGACATGATTTGAAAAGGCA
        a                                                a
TACACCTGTCTATATAAGGTCCCAGGGTTGACAGTGCATGTCAAAGCACAAACCAAGCATGA
               g
AGTCAAAGGAATTGTCTGTAAACCTCCGAGACAGGAATGTTTTAAGGCACAAGGCTGGGAA
  a                g         g     t   c g  c      a   t
GGTTACAGAAACATT-CTGCTGTTCTGAAAGTTCCAATGAGCAC-GTGGCCTCCATGATCTG
           g   t     c             g       a          c   ga
TA+GTGGAAGATGTTTGGAACCACAAGGACTCGTCCTACAGCTGGCCGGCCATCTAGGCTGA
  a                    c       t     g       t        a
GTGTTTGGGGGAGAAGGTCCTTAGTCAGGGAGGTGATCAATAACCCGATGGTCACTCTGTCT
   a c          g            g          t    a      a
GAGCTCAGCGTTCTTCTGTGGAGAGAGGAGAACCTTACAGAAGGACAACCATTTGTGCAGCA
      ------------------------------------------------------
ATCCACCAATCAGGCCAGTATGGCAGAGTAGCCAGACGGAAACCACTCCGCACCTGGAATTT
    g  t     gt  ta  t---    a-----  c-------c-  --     t
++GCCTAAAGACATATGAAACACTCTCAAACC+TAGGAAACTAAACTCTCTGGTCTGATGAG
ag a    cc-   c    gg      g   a  a    a   t  --g     a
ACTAATATTGAACTCTTTGGAGTGAATGCCAGGCATTACGTTTGGAGAAAACCAGTCAGCGC
     a                   ca     tg        at         a  c
TCATCACCAGGCTAATACCATCCCTACTGTTGGCAGCATCATGCTGTGGAGATGTTTTTCAG
 t    ta        c   c      a  -- a     gg  g    g
CAGCAGGAACTGGAAGACTAGTCAGGATAGAGGGAAAGATGAATGCAGCAATGTACAGAGAC
           a
ATCCTGAATAAAGACCTGCTGCAGAGTGCTCTTGACCTCAGACTGGGGCGGACGTTCATCTT
         g  a      t                            --------
TTAGCAGGACAATGACCCAAAGCACACTGCCAAAATATCAATGGAGTGGCTTCACAACAACT
ac                        c    -----------------------------
CGGTGAATGTCTTTGAGTGGCCCAGACAGAGCCCAGACCTAAATGGTATTGAACTTCTCTGG
------------------------------------       cc   a   a
AGAGATCTGAAAATGGCTGTACACCGTCACTTCCCATCCAACCTGATAGAGCTTGAGAGGTA
               t      t    g  g                      g   a
CTGCAAAGAGGAATGGCAAAAAT++++++++TCCCAAAGACAGGTGTGCCAAGCTTGTGGC
             a         aaatcaat aaa   t
ATCATATTCAAAAAGACTTGAGGCTGTAATCGCTGCCAAAGGTGCATCAACAAAGTATTGAG
             g      a          t-----  ca    a ------------
CAAAGGCTGTGAATACTGATGTACATGTGATTTTTCAGGTTTTTATTTGTAATAAATTTGC
                         aa                     a   c
AACAATTTCAAAAAATCTTTTTTCACATTGTCATTATGGGGTATTGTGTGTAGAATGTTGA
             t                           tc           t att
GGAAATAAATGA+ATTTA+++++ATCC+++++ATTTT+++++++++++++++++++++++
ttcc  ttttattt     ttta   ttattt     tcacctttctttggaaaggacagta
+++++++++++++ GGAAA++++++++++++++++++++++ AAGGCT+++++++++++++++
gagattgcagaca     gtattgggagcagagagaggggg    g tggcaaaggacttcga
+++++++++++++++++++++++GTGA+++++++++++++++++++++++++++++++++
gccgggaatcgaacttgggtcacc    gcaccatggtgctatatgtgcacttaaccactag
++++++++++++++++++++ CATAAAAAATGTGAAAAAGTGAAGCG+++++++++++++++
gctattggcaccgacttgtgagaatttga-g    tcaatc atcaatcaatccattaatc
+++++++++++++++ CTATGAATACTTTCCGGATGCACTGTA
tatctatctatccat    ct  ctg ctgtctgtctgtctgtctgtctgtctgtctg
```

3'
