# Supplementary material for: The zebrafish progranulin gene family and antisense transcripts
Source: BMC Genomics. 2005 Nov 8;6:156. doi: 10.1186/1471-2164-6-156 (PMC1310530; doi:10.1186/1471-2164-6-156)
Supplement: Additional File 9 — Primers used in the linkage group assignment of the zebrafish progranulin genes. Before applying to the LN 54 mapping panel, conditions for each primer combination were optimized by PCR with the use of zebrafish genomic DNA derived from the AB wild type strain. The authenticity and specificity of each amplicon was verified by sequencing after cloning into the pCRII plasmid. For the assignment to zebrafish linkage groups, each PCR amplification experiment was performed at least twice. [file 1471-2164-6-156-S9.pdf]

Progranulin-1 and Progranulin-2

map forward	5′ – ACT GTG TGT CCA GAC GG – 3′
map reverse1	5′ – CCA TCC CTG CAA CAC TG – 3′
map reverse2	5′ – TCT GGT GGA GGC GAA ATT – 3′

Progranulin-a

map forward	5′ – ATG TTG TGC AGT GTG AAG GAC – 3′
map reverse	5′ – CAT CAG CAG ACA GGA GAA CTC – 3′

Progranulin-b

map forward	5′ – AAC GCA TGC ACT GCT CTG ATC – 3′
map reverse	5′ – AGT TCC CTG ATA ACA GCA GTG – 3′
