# Supplementary material for: The zebrafish progranulin gene family and antisense transcripts
Source: BMC Genomics. 2005 Nov 8;6:156. doi: 10.1186/1471-2164-6-156 (PMC1310530; doi:10.1186/1471-2164-6-156)
Supplement: Additional File 2 — Genomic architecture of the zebrafish grn1 gene. Exons (1–5) and deduced amino acid sequences are shown in uppercase letters and are boxed, while introns (A–D) and flanking sequences are shown in lowercase. 5' extension of exon-1 is based on a deposited EST sequence (accession number BG884011) and 5' RACE. A potential TATA box in the promoter region is in bold and italics. The translation initiation codon (ATG) and polyadenylation signal sequence (AATAAA) are in italics. The EcoRI restriction site (gaattc) used for cloning the genomic fragments for this gene is located in intron C, and is underlined and bold. An identical exonic architecture was found for grn2 (data not shown). [file 1471-2164-6-156-S2.pdf]

```
  cgggcagccattgaagagaagatcaaatcggggtgatgtgctcaaatttgcgtgtgccagtttaaagacgtgcagctgcattctgaacaaaatgcag
  tcgagaaatagaacactgactcaccccaaaataaagggcatcgcagtagtccaagtgggatataaaagcatggattactgttttaaagtgactgtgt
  gataaaatatgctcaattttggccagttgtctcaaagtgaaagaagctagagcgaacaacagaccgtttgccatatgatggcaacagaacaacagat
  ctttgccatgatgacagtaaataatattttactagatacttttcaagacacttctatacagcttaaagtgacaattaaaggcttaactaggttaatt
  agggtaactaggcaggttagggtgattaggcaagttattgtaaaatgatgatttgttctgtagactacaggaaaaaaattgcttaatggggctaata
  attttccttaaaatggtattaaaaaattaaaaactgcttttattctaaccgaagaaaaacaaataagactttcttcagaacaaaaaatattatcaga
  catactctaaaaattaccttgcctgtactgtgtgtgtgtatatatatatatgtatgtgagaaggaactactgactaaagtgacttatcacatcacca
  tgctttcatatccttatatacattactttcattcaaacacttctccaagttcatgatcctgtttctaaatgccatgggtcacaagataactgATAAG
  ATTTCCTGTTAGTATAAATCTCAACAACAAATCTGATGAAGTTTCCAGTAATTGCACTGGGAGGAGTCTGTCTACAAGTATAAGAAGTACAGCAGAG   exon 1
  AATTACCGGACAGAACGACACAGACAGCAGCAGAAGCATCCCGAACAGGAGGAAACCCAGCCAGCAAGgtaaatgttattacatcactctcagctaa
  tgtattatgggtaaagacaactaattgtaatgggtagtattgtatacagtcagtgcacagcataaatgagggcaaccctctttaaaagatgaatctc
  tttatcatttctcagtaaatatagcgtcaatatattttgatccagactttactttggttctgtcaaatgtaatgtatacacacaagtatattattgt
A attgacctgattctttattgtggaagtgcgcttattttgcaattgttttagaacttccgattcagtagtctatggtagaaatgactacgaataataa
  accacagaaaacggtcaaactactccctctacaaacaagtgtttgcagaaaaagcaaaatcaaataataagaaaatatcagtttgcaacatcaagca
  gcagaactagctgtttttaacttctaaaaatgaatggaagtaaataagaccggaagtctcaagttaaaacgattcaaatggctgtgcccgctcttac
  gcgaagaataaggtcaatagcatcctatagaaagttttaatttaaaagagagataagtcggggtgtactcatttataatgggcagtgtcctaaatta
  aactaaaataaaatataataaaatagaaaacaaaaatgcgctgataaaataaaaaaagataaaatatacctactactctcctatttcacattttcag
  ATGTTCCCAGTGTTGATGTTACTCATGGCGGCTCTGGTGGCTGCAGATGAGCCTCTGCTTGATCTGTCTATCCCGGTGGAGACTGTAGACACCTCTG
   M  F  P  V  L  M  L  L  M  A  A  L  V  A  A  D  E  P  L  L  D  L  S  I  P  V  E  T  V  D  T  S     exon 2
  CCTCTGTGATTCACTGCGATGCTCAAACTGTGTGTCCAGACGGAACAACATGCTGTCTGAGTCCATATGGCATATGGTCATGTTGTCCTTACTCAAT
  A  S  V  I  H  C  D  A  Q  T  V  C  P  D  G  T  T  C  C  L  S  P  Y  G  I  W  S  C  C  P  Y  S  M
B GgtaacagaatctcgagttaatatgtttctactcatagttacagggttaacctttgttcactctttcctctgcttcagGGTCAGTGTTGCAGGGATG
                                                                                  G  Q  C  C  R  D     exon 3
  GGATTCACTGTTGTCAACATGGATATCGCTGCGATTCCACCTCCACCCGATGCCTGCGGGGCTGGCTGACGCTGCCATCGTCCTTCCAGAAGGCCAC
  G  I  H  C  C  Q  H  G  Y  R  C  D  S  T  S  T  R  C  L  R  G  W  L  T  L  P  S  S  F  Q  K  A  T
  CAGAACCTTCCAGAAAGATCAGgtatggcagaccactgtaaaaataaaatatgtaaataaaatagtgtggtgcaaatactaattgcattcaaaataa
    R  T  F  Q  K  D  Q                                                             aagactaaaaagtttcttt
  gcaaaacacacacttacataaaaacaaaattatacaacatttttttgcatcaatatttaaattgatatatttaaggaaggaaattttcacagtatgt
  ccgataatatttattcttctggaaaaagtctcatttgttccatttcggctagaataaaagcactttttaaatttaaaagaaaaaaattttgggtcaa
  aattattagccccttttaagctatatttttttcgttagtctacagaacaaaccgtcactccgttaaaaataaaaaaataaaaataaaaaggggggcct
  tataattctgacttcaactgtatacacagtacacacacacacacacacacacacacacacacacgcacacacacacacatacacacacacacacaca
  tatatatatatatatatatatatatatatatatatatatatatatatatatatatatattttttttttttttttttttttttgggatgcgattaactg
  tgattagtctttgcccagcactaaaataaatgtaaaaaaatctaatataaacatttctaattcaaataatatattaagatgataatgatatataaat
  attaaaaaatattactaaagttagacatgcagatattcaataatacacagtgtgatattgaacatgcatgatattgttattgttaaaatactgtgag
  taaatattcattattcctttttatcacatcaattatcacgacaagaaaatgcaataattaccatcgtatccttgaatgcactacatttatatttaag
  gtattttttattgtttcaaaatgtattttgtttcagtgttactcaatatttttaagtaattgtctgtgaactgcagtcatttctaagttaaaatagt
  ttctattgtatacctatgtgctagtaaagcatcttgtgctacagtgacctacatgacaatctttaactctcgatgtcgtatgactttgaatcaccac
  ttcccttggcaataaaacaccgtttcaacactttcaagctgcaaacaatgaaacaatgtacaataaaagacatgtgactttcaagcagtcaaaggcc
  acagtcatgttaaaagttaaaatcagttgttaattcaactttattagtgcttgaagacattataagtgcttgatttaggagaatagcaaatattgca
C tctagaaaaaactgttataaagatttggtcaaatgcacactgcagtgtatcattcattcattttcttgtcggcttagtcccttattaatgcggggtc
  gccacagcggaatgaaccaccaacttatccagcaagtttttacgcagcggatgcccttccagccgcaacccatctctgggaaacatccccttacaca
  atcacgcacacacttacagacaatttagccttcccaatcccccctgtaccacatgtctttggactgtggggaaaaccggagcacccggaggaaaccc
  acgcaaacccaagaacatgcaaactccacacagaaacgccaactgagccgaggttcgaaccagcaacttcttgctgtgaggtgacagcactacctac
  tgcgccactgcctcgccccactgcagtgcatacaaaaccaaaatctacaaatagctcttgatttgaaaagacactgtagttgataaaacaaagaaca
  acagggttttaattacgtataatttttaaggaacaaaaccatcaaagcaaagtttaactcaaacccataaatgacaaagcgaaacattgaattcttg
  actgatttcattcatgaactggattgcttaagatgctgcacattatgtttttgaaagtacgatgtgatcctgaattaatgtctatgttcattatttt
  tgtttgataatgtaatccctacacctaaacccaaccataactgtatttcccaaatcagaagggaaaaatagttgcataacaatcatgtaaaagtgca
  taaacctaaccattagcctaaatttaacatgaacggtaaacagttatcacttcattttgtaattgttattggctgattggaatgttgttccaggatc
  aacatagatgttaagcacatgtcccacttggataaatcaggttggcgtttaaggcaaagatgattactaatcttaaaaataagktgactttgcagca
  cgtcaacaccacattctgtctgtggtagtttgaattatgtgagctgcaatcatttaaattggtcagactaatgtaattcttgtgaatgtaaatttag
  ACCCACGCTGAGACTGTCCAATGTGAAGGAAATTTCTACTGCCCGGCTGAGAAGTTTTGCTGCAAGACTAGAACTGGCCAGTGGGGCTGCTGCAGCG   exon 4
   T  H  A  E  T  V  Q  C  E  G  N  F  Y  C  P  A  E  K  F  C  C  K  T  R  T  G  Q  W  G  C  C  S
D gtaaaacagacttcatatcactgtattaataagcatggctaatcaagaagcataatataatacaattcacttacaacctgtatttctatttttcata
  gGGTTGGAGTTGTAAGCAAACAACCACTGTTACACTGGTTCACCTCTTACTGAAGACTGTCAAAATGCAAGAGGCTCCAAACTTCATTCATGTTTCA
   G  L  E  L  *                                                                                       exon 5
  GGCTATATTCTTACATCCAGGCTGAATTAGTGTAAAATAAAAACTGATAGAATTACTGTCAAAATGCACCGTCATTTAATAAACATTATTAATTTGG
  GGGAAAAgaaattgtgttcttattcattcattcatagtatttttatgaagtaatcagtgattgtgaatgaatttaatgcactgtaaagcccaacagt
  ccactttatcaaatgaattgagtgaaaga
```
