# Supplementary material for: The zebrafish progranulin gene family and antisense transcripts
Source: BMC Genomics. 2005 Nov 8;6:156. doi: 10.1186/1471-2164-6-156 (PMC1310530; doi:10.1186/1471-2164-6-156)
Supplement: Additional File 10 — Sequences of primers and predicted sizes of PCR amplicons. All primer pairs are located on consecutive exons of their corresponding gene. [file 1471-2164-6-156-S10.pdf]

gene	primers	size (bp)	annealing T
grna	FOR: 5′- CTCAACTCACTCACATCCGC - 3′	301	60
	REV: 5′- GTTTATAGAGTTAGGGCTCG - 3′		
grnb	FOR: 5′- ACAATGGTGCGTGCAGCTTTC - 3′	381	60
	REV: 5′- GCACACGGCATTTTTCATAGG - 3′		
grn1+2	FOR: 5′- ACAGACAGCAGCAGAAGCATC - 3′	409	60
	REV: 5′- TTCACATTGGACAGTCTCAGC - 3′		
grn1	FOR: 5′- GGTGGAGACTGTAGACAC - 3′	—	
grn2	FOR: 5′- GATGGAGACTGAAGACGT - 3′		
grn1	REV1: 5′- TCGGGTGGAGGTGGAATC - 3′	190	61
grn2	REV1: 5′- TCTGGTGGAGGCGAAATT - 3′		63
grn1	REV2: 5′- GCTTACAACTCCAACCCG - 3′	372	61
grn2	REV2: 5 - GCTTACAACTCCAACTCA - 3′		63
ASgrn1-2	FOR: 5′- TTAACTTGTCGAGCGTTTCCC - 3′	556	60
	REV: 5′- GCAGACCACTAATACTCTCCT - 3′		
actin	FOR: 5′- ATGGATGATGAAATTGCCGC - 3′	253	60
	REV: 5′- TGTCATCTTTTCCCTGTTGG - 3′		
ASgrna 1	FOR: 5′- GCACTAAGCAGTAACGATGGTC - 3′		
2	REV: 5′- ACTGGAGCAACTGGGTCAACTG - 3′	342	61
3	REV: 5′- GCTGTTGGTAACTCTATCCTCG - 3′	478	61
